# CRATER tumor niches facilitate CD8^+^ T cell engagement and correspond with immunotherapy success

**DOI:** 10.1016/j.cell.2025.09.021

**Published:** 2025-10-17

**Authors:** Aya Ludin, Georgia L. Stirtz, Asaf Tal, Ajit J. Nirmal, Kathleen L. Pfaff, Michael Manos, Naomi Besson, Nebiyat Eskndir, Billie Porter, Stephanie M. Jones, Hannah M. Faulkner, Qiyu Gong, Sophia Liu, Irving Barrera, Lijian Wu, Cecilia Pessoa Rodrigues, Aditi Sahu, Elizabeth Jerison, Joao V. Alessi, Biagio Ricciuti, Douglas S. Richardson, Jodi D. Weiss, Hadley M. Moreau, Meredith E. Stanhope, Alexander B. Afeyan, James Sefton, Wyatt D. McCall, Emily Formato, Song Yang, Yi Zhou, David P. Hoytema van Konijnenburg, Hannah L. Cole, Miguel Cordova, Liang Deng, Milind Rajadhyaksha, Stephen R. Quake, Mark M. Awad, Fei Chen, Kai W. Wucherpfennig, Peter K. Sorger, F. Stephen Hodi, Scott J. Rodig, George F. Murphy, Leonard I. Zon

**Affiliations:** 1 Harvard Stem Cell and Regenerative Biology Department, Harvard University, Cambridge, MA 02138, USA; 2 Department of Dermatology, Brigham and Women’s Hospital, Harvard Medical School, Boston, MA 02115, USA; 3 Ludwig Center at Harvard, Boston, MA 02115, USA; 4 Center for Immuno-Oncology, Dana-Farber Cancer Institute, Boston, MA 02115, USA; 5 Broad Institute of MIT and Harvard, Cambridge, MA 02139, USA; 6 Ragon Institute of MGH, MIT and Harvard, Cambridge, MA 02139, USA; 7 Department of Cancer Immunology and Virology, Dana-Farber Cancer Institute, Boston, MA 02115, USA; 8 Dermatology Service, Memorial Sloan Kettering Cancer Center, New York, NY 10021, USA; 9 Department of Physics, University of Chicago, Chicago, IL 60637, USA; 10 Lowe Center for Thoracic Oncology, Dana-Farber Cancer Institute, Boston, MA 02115, USA; 11 Harvard Center for Biological Imaging, Department of Molecular and Cellular Biology, Harvard University, Cambridge, MA 02138, USA; 12 Stem Cell Program and Division of Hematology/Oncology, Boston Children’s Hospital, Boston, MA 02115, USA; 13 Division of Immunology, Boston Children’s Hospital and Department of Pediatrics, Harvard Medical School, Boston, MA 02115, USA; 14 Department of Bioengineering and Applied Sciences, Stanford University, Stanford, CA 94305, USA; 15 Chan Zuckerberg Biohub, San Francisco, CA 94158, USA; 16 Laboratory of Systems Pharmacology, Department of Systems Biology, Harvard Medical School, Boston, MA 02115, USA; 17 Department of Medical Oncology, Dana-Farber Cancer Institute, Boston, MA 02115, USA; 18 Parker Institute for Cancer Immunotherapy, Boston, MA, USA; 19 Department of Pathology, Brigham and Women’s Hospital, Harvard Medical School, Boston, MA 02115, USA; 20 Howard Hughes Medical Institute, Harvard Medical School, Boston, MA 02115, USA; 21 These authors contributed equally; 22 Lead contact

## Abstract

**SUMMARY:**

T cell-mediated tumor killing underlies immunotherapy success. Here, we used long-term *in vivo* imaging and high-resolution spatial transcriptomics of zebrafish endogenous melanoma, as well as multiplex imaging of human melanoma, to identify domains facilitating the immune response during immunotherapy. We identified cancer regions of antigen presentation and T cell engagement and retention (CRATERs) as pockets at the stroma-melanocyte boundaries of zebrafish and human melanoma. CRATERs are rich in antigen-recognition molecules, harboring the highest density of CD8^+^ T cells in tumors. In zebrafish, CD8^+^ T cells formed prolonged interactions with melanoma cells within CRATERs, characteristic of antigen recognition. Following immunostimulatory treatment, CRATERs expanded, becoming the major sites of activated CD8^+^ T cell accumulation and tumor killing. In humans, elevation in CRATER density in biopsies following immune checkpoint blockade (ICB) therapy correlated with a clinical response to therapy. CRATERs are structures that show active tumor killing and may be useful as a diagnostic indicator for immunotherapy success.

**In brief:**

CD8^+^ T cells preferentially engage tumor cells in pockets termed CRATERs, which are rich with antigen presentation molecules and breach into the tumor mass from the stromal compartment. CRATERs expand and become the major site of tumor killing during immune-stimulatory treatment, and their expansion parallels clinical benefit in patients undergoing immunotherapy.

## Graphical Abstract

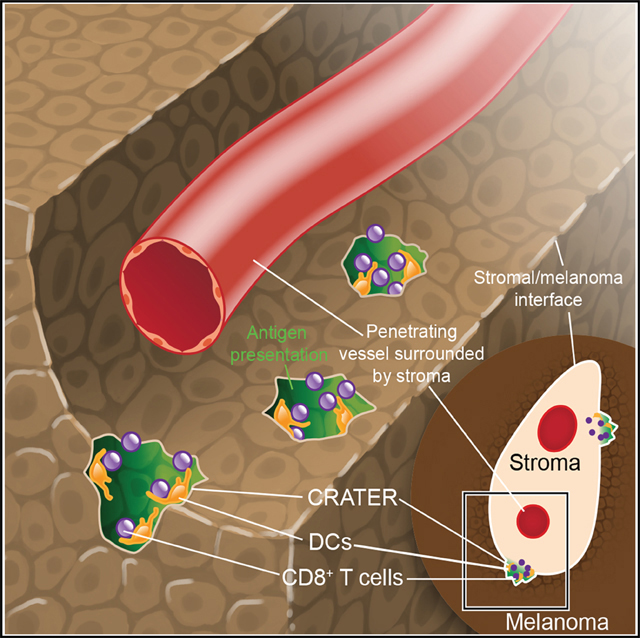

## INTRODUCTION

Immune checkpoint blockade (ICB) cancer therapy, re-activating CD8^+^ T cells against tumors, has significantly increased patients’ life expectancy and survival.^1,2^ ICB initiates CD8^+^ T cell infiltration and results in necrosis, fibrosis, and shrinkage. Markers indicating T cell infiltration and activation have been associated with positive treatment outcomes.^3,4^ However, what facilitates local tumor killing by the infiltrating CD8^+^ T cells is not yet known. Human melanoma driver mutations, such as BRAF(V600E), induce endogenous melanoma growth in zebrafish,^5^ with shared genomic and morphological characteristics of human melanoma,^5–7^ and can be generated in zebrafish with a highly conserved immune system.^8^ To date, obtaining a 3-dimensional (3D), dynamic view of tumor-immune interactions *in vivo* has been challenging, limiting our understanding of the nature of the anti-tumor immune response within an intact tissue microenvironment. The zebrafish model provides a unique opportunity to perform such imaging of immune cells as they interact *in situ* with endogenous tumors. Here, we created a *cd8α*:EGFP transgenic fish and adapted a water flow system^9^ to allow live time-lapse confocal imaging for over 15 h, providing capture of slow or rare events. This enabled us to follow infiltrating CD8^+^ T cells as they move through the 3D architecture of endogenous tumors. We found that rather than diffusely patrolling the tumor surface, CD8^+^ T cells aggregate in pockets on the melanoma border, forming prolonged interactions with melanoma cells. The melanocytes lining the pockets locally present elevated beta-2 microglobulin (B2M) levels that retain the CD8^+^ T cells in them for many hours. We termed such a pocket cancer region of antigen presentation and T cell engagement and retention (CRATER). CRATERs exist in both zebrafish and human melanomas and become major sites of tumor killing upon immune-stimulatory CpG oligodeoxynucleotides (CpG ODN) therapy in zebrafish. Highly multiplexed imaging of human melanoma shows similar major histocompatibility complex (MHC)-high CRATERs, which preferentially harbor CD8^+^ T cells as well as PD-L1^+^ and CD163^+^ dendritic cells (DCs). CRATER numbers increase in patients responding to ICB treatment, whereas patients with progressive disease (PD) post treatment demonstrated disseminated CD8^+^ T cell infiltration throughout the tumor parenchyma and few CRATERs. Our study thus identifies physical regions, CRATERs, on the tumor-stromal interfaces in which the tumor is highly recognizable to CD8^+^ T cells. These regions facilitate the anti-tumor immune response upon treatment.

## RESULTS

### Generation of CD8^+^ T cell reporter zebrafish

To observe CD8^+^ cell interactions with melanoma *in vivo*, we created a transgenic (Tg) zebrafish line that labels cells expressing *cd8a*. We cloned a region upstream of the *cd8a* zebrafish gene, containing two open chromatin areas identified in sorted lck^+^ T cells by assay for transposase-accessible chromatin using sequencing (ATAC-seq) (Figure S1A). This clone was used to drive cytoplasmic EGFP expression in *cd8a*-expressing cells. EGFP expression was evident in the zebrafish thymus at 1 week and was prominent in the juvenile fish thymus at 6 weeks post fertilization (Figure S1A). EGFP labeled a distinct population of lck^+^ cells of 10-μm diameter in the fish major lymphoid organs (Figure S1B). RNA sequencing (RNA-seq) of sorted EGFP^+^ lymphocytes from juvenile fish thymi showed a gene expression profile characteristic of CD8^+^ T cells. *cd8a*, *cd8b*, pan-T cell markers (*lck* and *cd3eap*), T cell receptor (TCR) signaling molecules (*zap70*), and transcription factors required for CD8^+^ T cell development (*gata3* and *runx3*) were expressed, but not *zbtb7b*, which is required for CD4^+^ T cell development^10^ (Figure S1C). Low levels of *cd4* expression were detected, likely due to CD8^+^/CD4^+^ double-positive T cells that exist in juvenile fish thymi.^8^ The cd8a:EGFP^+^ population was markedly reduced in prkdc^(−/−)^ zebrafish that lack T cells^11^ (Figure S1D). Taken together, the Tg(cd8a:EGFP) zebrafish reports CD8^+^ T cells.

Tg(cd8α:EGFP) fish also labeled a population of myeloid cells, 50–70 μm in diameter, with a distinct dendritic shape (Figures S1E and S1H). RNA-seq of sorted cd8a:EGFP^+^ myeloid cells from melanoma revealed gene expression resembling murine CD8^+^ DCs that, in mammals, cross-present antigens with high efficiency to CD8^+^ T cells.^12^These cells expressed the zebrafish myeloid markers *mpeg* and *mfap4* but not the neutrophil marker *mpx* (Figure S1G). Transcription factors important for CD8^+^ DC development (*id2*, *batf3*, and *irf8*, but not *irf4*)^13^ or marking classical DCs (*zbtb46*)^14^ were highly expressed (Figures S1F and S1G). *cd8a*, but not *cd8b*, was expressed in this population (Figure S1F), characteristic of CD8^+^ DCs.^12^ The pan-T cell marker *lck* was not expressed (Figure S1F), excluding T cell contamination. These CD8^+^ cells presented AlexaFluor 594-conjugated ovalbumin (OVA) *in situ* when injected intraperitoneally, demonstrating the ability to uptake antigens (Figure S1H). This indicates that, in addition to CD8^+^ T cells, the Tg(cd8α:EGFP) fish marks a CD8^+^ myeloid population that resembles murine CD8^+^ DCs.

### T cells infiltrate melanoma tumors in zebrafish

**Figure 1.**
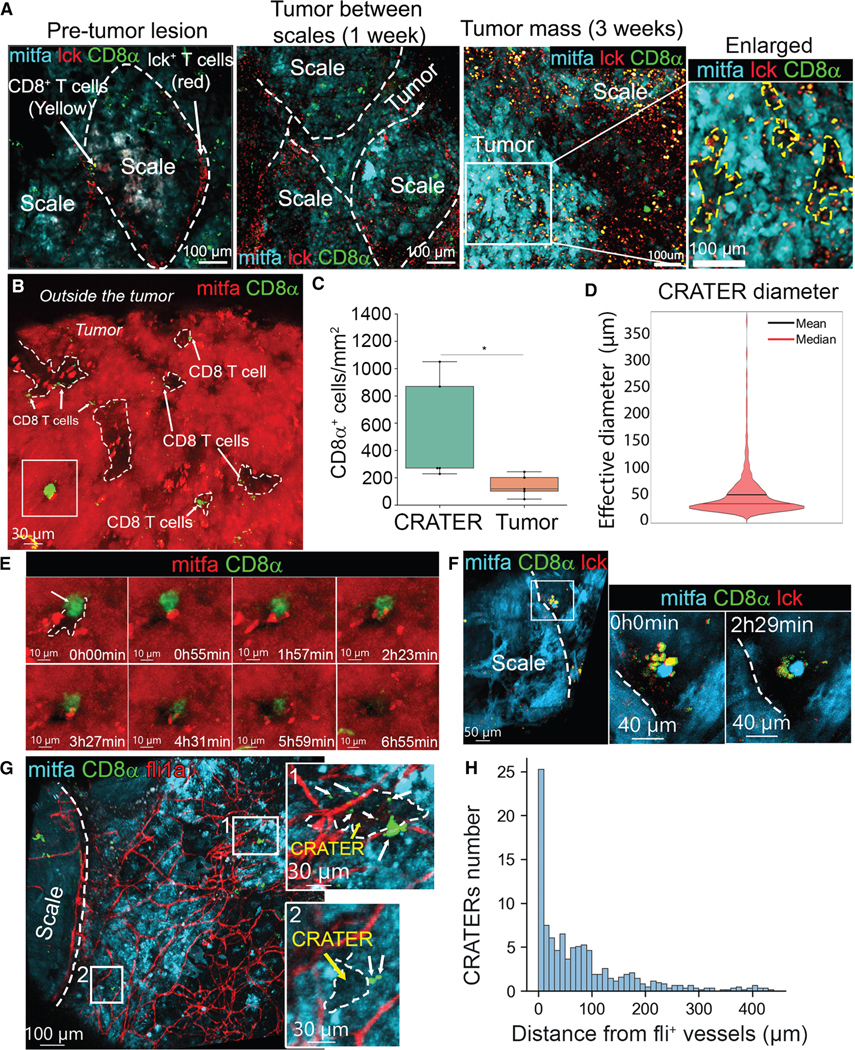
CD8^+^ T cells preferentially locate in CRATERs on the melanoma surface (A) Longitudinal imaging of a mitfa:BFP tumor across stages of development in a Tg(cd8a:EGFP;lck:mCherry) fish. Dashed white lines: scale edges. Left: radial growth of melanoma below the scale. Middle: same tumor after 1 week. Right: same tumor after 3 weeks. Area in rectangle enlarged. Yellow dashed lines: CRATERs. (B) 3D projection of a melanoma tumor in a Tg(cd8α:EGFP) zebrafish (representative of over 100 tumors). Arrows: CD8^+^ T cells. White dashed lines: CRATERs. (C) CD8^+^ cell density (cells per mm^2^) in CRATER vs. tumor area (*n* = 5 fish, whiskers represent 1.5× the interquartile range. Mann-Whitney U test, *p value = 0.015). (D) CRATER diameter size (μm) distribution in untreated tumors (*n* = 3 fish/265 CRATERs). (E) Snapshots from time-lapse imaging of area at rectangle in (B). Dashed line: CRATER. Time is h:min. (F) CRATER at tumor-infiltrated scale edge. Dashed line: scale edge. Time is hours:minutes. (G) Representative 3D image of a tumor in Tg(cd8α:EGFP; fli1a:dsRed) zebrafish. Rectangle areas: enlarged to the right. Dashed lines: CRATERs. White arrows: CD8^+^ T cells. CRATERs emerge adjacent to the fli1a^+^ blood vessel. CD8^+^ T cells interacting with: (1) blood vessels and (2) tumor cells. (H) Number of CRATERs at distances from fli^+^ blood vessels (*n* = 3 fish, 630 CRATERs). See also Figures S1 and S2.

We next visualized immune infiltration into zebrafish cutaneous melanomas. We generated melanomas using two approaches. The first utilizes Tg(cd8a:EGFP;BRAF^V600E^/p53^null^/nacre^null^) zebrafish that harbor melanoma-driving mutations but lack *mitfa*, which is necessary for melanocyte formation. Tumors are created by re-introducing the *mitfa* minigene along with a fluorophore, using the MiniCoopR vector^6^ injected into embryos. This creates endogenous tumors, as widely reported by our lab.^6,7^ The second approach introduces the BRAF^V600E^ mutation *de novo*, along with melanocyte-specific CRISPR of p53^15^ and tyrosinase. This approach produces low-pigmented tumors in lower frequency but enabled melanoma generation in endothelial reporting or Tg(cd8a:EGFP;lck:mCherry) zebrafish. To explore the nature of all T cells infiltrating the zebrafish melanoma tumor, we used a transgenic zebrafish that reports all T cells through mCherry expression driven by the zebrafish promoter of the *lck* gene.^16^ Single-cell RNA-seq (scRNA-seq) showed that lck^+^ T cells infiltrating zebrafish tumors (Figures S1I and S1J) contain CD8^+^ and CD4^+^ T cells, including Foxp3^+^ regulatory T cells (Tregs) (Figures S1K and S1L). We visualized T cell infiltration as the tumor developed over time in the same fish by longitudinal imaging (Figure 1A). In healthy fish skin, most of the lck^+^ T cells are CD8^−^ (Figure S1M). During radial growth of early melanocytic (pre-tumorous) lesions, lck^+^ T cells, with very few CD8^+^ cells, retained their normal distribution along the scale edges, creating tessellated structures (Figure 1A, left). These structures were reported to serve as peripheral immune surveillance sites that functionally resemble lymph nodes.^17^ As the tumor protrudes from the scales to form a mass, the tessellated pattern is disrupted (Figure 1A, middle) and ultimately disappears, while multiple CD8^+^ T cells infiltrate the tumor (Figure 1A, right). At this stage, we noted that the infiltrating CD8^+^ T cells are enriched in areas devoid of mitfa^+^ melanocytes (Figure 1A, right enlarged).

### CD8^+^ T cells localize to CRATERs and engage with tumor cells at the melanoma surface

To study this phenomenon, we quantitatively analyzed 3D confocal images of endogenous zebrafish melanoma. We found that CD8α^+^ cells preferentially localized to mitfa^−^ areas that form pockets lined by melanoma cells (Figure 1B)—here termed “CRATERs”—in all melanoma tumors imaged during this study (>100 fish). We computationally segmented the CRATERs to determine CRATER size (Figure S2A) and interaction with CD8^+^ T cells (Figure S2B). We found that CD8^+^ T cell density was significantly higher in CRATERs compared with the rest of the tumor surface (Figure 1C), with the majority of CD8^+^ T cells found within 5 μm of a CRATER (Figure S2C). This suggests that CD8^+^ T cells preferentially localize to CRATERs. The CRATERs measured ∼50 μm in diameter (Figure 1D) and constitute ∼12% of an untreated tumor surface (Figure S2D). To visualize CD8^+^ cell activity within CRATERs, we adapted a water flow system^9^ to keep zebrafish alive and anesthetized under a confocal microscope and tracked 3D cellular dynamics for 15–20 h via time-lapse imaging. CD8^+^ T cells lingered in CRATERs and engaged with melanoma cells for many hours (Figures 1E and S2E; Video S1). This was evident within the tumor mass and in tumor-infiltrated scale edges, where clusters of CD8^+^ T cells directly engaged tumor cells within CRATERs (Figure 1F; Video S2) in a manner resembling tumor recognition and killing, as seen *in vitro*.^18^

We evaluated CRATER proximity to tumor vasculature by imaging tumors grown in Tg(flk1:EGFP; fli1a:dsRed) zebrafish, marking blood vessels.^19^ scRNA-seq of sorted fli1a^+^/flk^+^ and fli1a^+/^flk^−^ cells showed that fli1a^+^/flk^−^ endothelial cells correspond to venous and lymphatic cells and fli1a^+/^flk^+^ represent a mix of venous and arterial endothelial cells (Figures S2G–S2J). CRATERs emerge at the perivascular area, adjacent to fli^+^/flk^−^ and fli^+^/flk^+^ vessels (Figures 1G and S2F). Most CRATERs were found within 100 μm of a blood vessel, many at 10-μm distance (Figure 1H). CD8^+^ T cells in the CRATERs contacted either fli1^+^ vessels (Figure 1G, area 1) or melanoma cells (Figure 1G, area 2). Though not specific to CRATERs, mpeg^+^ cells, marking myeloid cells,^20^ were found in CRATERs (Figure S2L, area 1). In addition, we noted CD8α^+^/mpeg^+^ cells over CRATERs in untreated tumors (Figure S2L, area 2). These cells were larger than CD8^−^/mpeg^+^ cells and had a dendritic shape (Figure S2L). As we found CD8^+^ myeloid cells to express the DC-specific marker *zbtb46* (Figure S1F), we stained zebrafish tumors for *zbtb46* expression using RNAscope. Though rare, we found *zbtb46*-expressing CD8α^+^/mpeg^+^ cells in areas corresponding to CRATERs (Figure S2M), indicating a possible DC identity. However, the nature of these cells *in situ* needs further verification. Another CRATER characteristic was the presence of fine collagen fibers extending into CRATERs from the capsule surrounding the zebrafish tumor, seen using second harmonic generation^21^ (Figure S2K).

### The CRATERs are sites of elevated B2M levels and antigen recognition for CD8^+^ T cells

**Figure 2.**
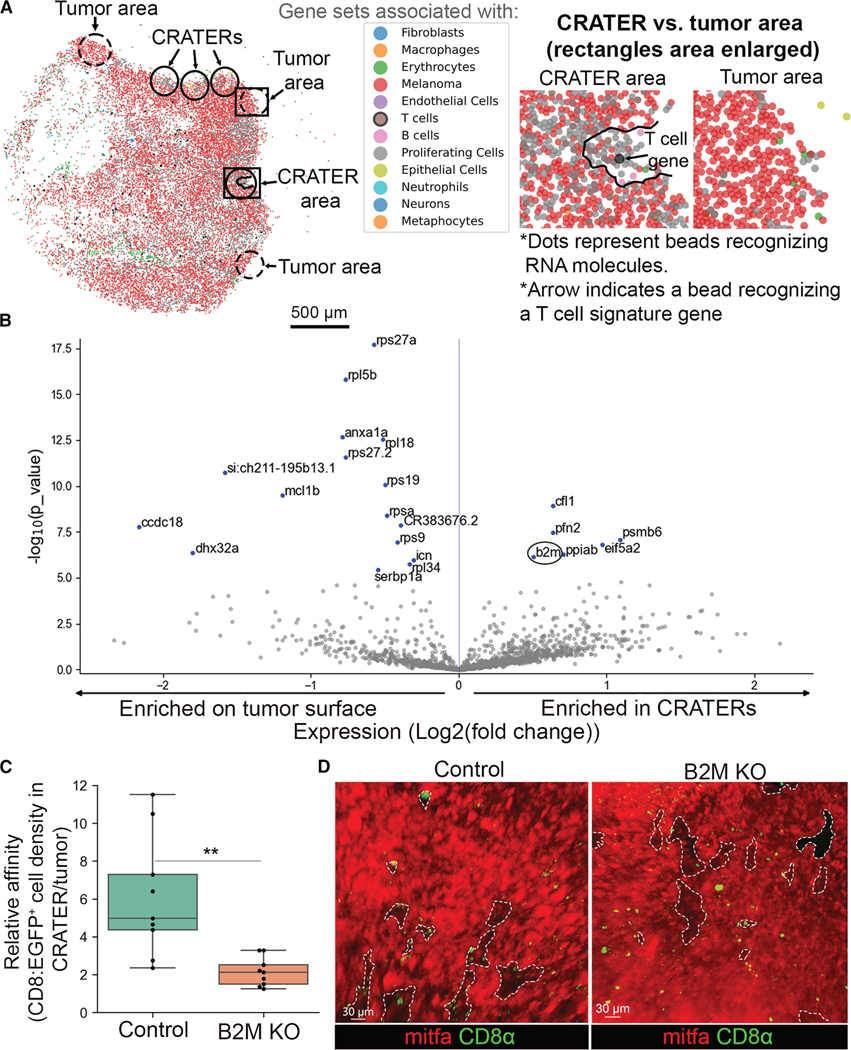
CRATERs are sites of elevated *b2m* expression that retain CD8^+^ T cells in them (A) Left: Slide-seq gene expression map of zebrafish melanoma tumor. Black circles: CRATER areas. Dashed black circles: non-CRATER areas. T cells that have entered the tumor can also be noted. Right: rectangle areas, enlarged, showing CRATER and non-CRATER areas. (B) Differential gene expression in CRATER vs. non-CRATER-associated *mitfa*^+^ areas. (C) CD8^+^ cell density in CRATER/non-CRATER tumor surface, in WT and melanocyte-specific B2M-depleted tumors. (Image quantification. *n* fish = control-4, B2M KO-9. Whiskers represent 1.5× the interquartile range. *t* test. *p value = 0.003). (D) 3D representative image of B2M intact (control) vs. depleted melanoma. White dashed line: CRATERs. See also Figure S4.

To further characterize the CRATER areas on the melanoma surface, we performed high-resolution spatial transcriptomics (Slide-seqV2)^22^ on zebrafish tumors. This yielded a gene expression map across the tumor landscape. We identified, at the rim of the tumor, areas lacking melanoma gene expression, creating pocket-shaped areas akin to CRATERs, which occasionally contained RNA for genes characteristic of T cells (Figure 2A). We performed differential gene expression analysis, comparing areas of melanoma gene expression at the projected CRATER areas’ border to areas not associated with CRATERs at the tumor surface (Figure 2A). CRATER-related areas presented elevated expression of *b2m* (Figure 2B), encoding B2M. B2M is a core molecule of the MHC class I complex, enabling tumor recognition by CD8^+^ T cells, which leads to sustained CD8^+^ T cell-tumor interactions,^23^ similar to those described above. To examine whether B2M mediates CD8^+^ T cell retention to CRATERs, we inserted a gRNA targeting *b2m* into a melanoma-specific CRISPR vector^24^ that was injected into Tg(cd8α: EGFP) embryos along with melanoma-generating vectors (described above), inducing melanoma-specific B2M-depleted tumors (Figure S3A). The tumors presented a significant reduction in the ratio of CD8^+^ T cells within CRATERs to those embedded in the tumor, compared with wild type (WT) (Figure 2C), as multiple CD8^+^ T cells were embedded in the tumor mass rather than aggregated within CRATERs (Figure 2D). This indicates that impaired melanoma recognition leads to either reduced migration of CD8^+^ T cells to CRATERs or decreased duration of CD8^+^ T cells dwelling within CRATERs. The overall CD8^+^ T cell numbers and CRATER coverage (i.e., the percent of tumor surface accounted for by CRATERs) were not significantly affected (Figures S3B and S3C). In this model, B2M is only inactivated in melanocytes and melanoma cells. B2M is expressed by the other components of the tumor microenvironment, which could be causing T cell infiltration to the tumor. In addition, because antibodies against zebrafish CD8 are unavailable, we do not exclude the potential difference in gene expression vs. protein expression, which may affect the measured absolute cell numbers. B2M loss reduced CD8^+^ T cell affinity to the CRATERs. Together with the characteristic long-term interactions of CD8^+^ T cells with melanoma in CRATERs, this indicates that CRATERs are sites where CD8^+^ T cells recognize tumor cells and engage them.

### Activated CD8^+^ cells accumulate in enlarged CRATERs following immune stimulation

**Figure 3.**
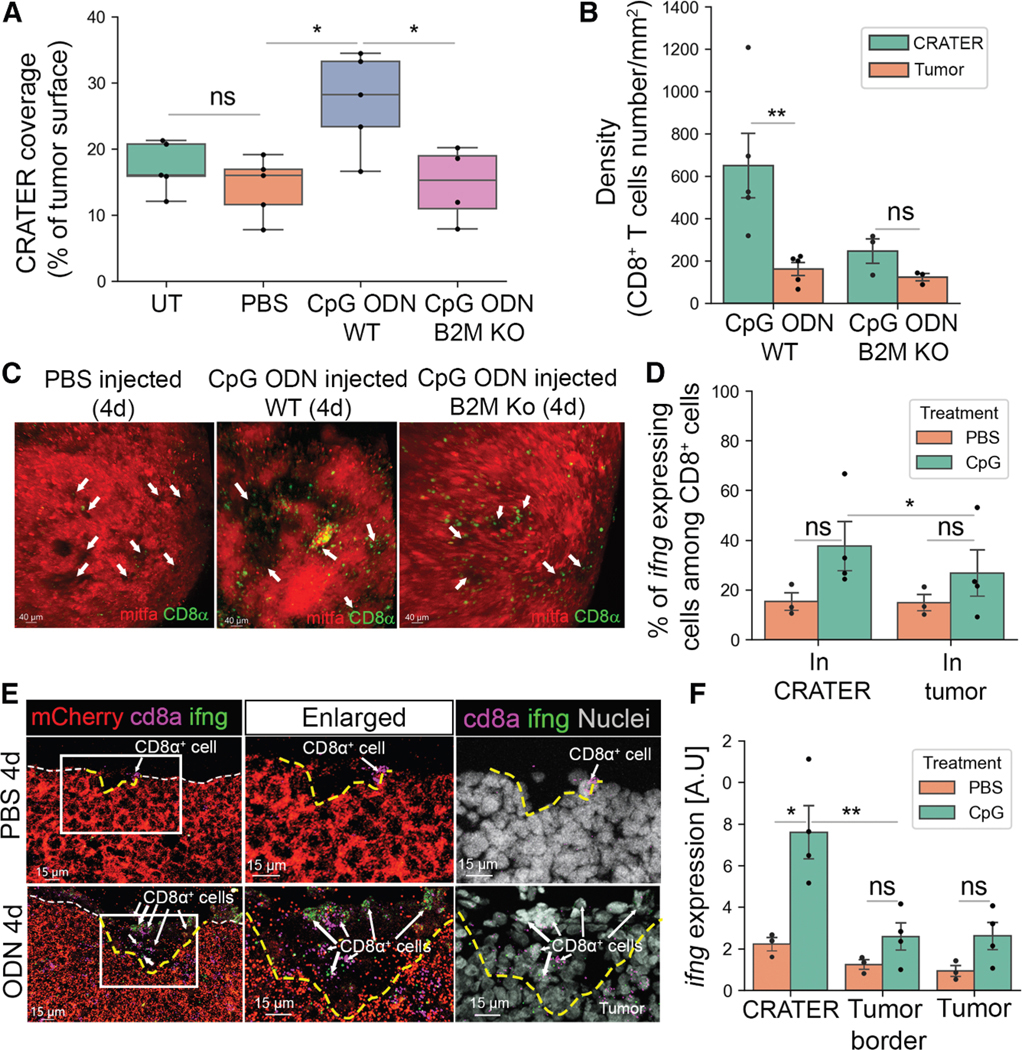
Activated CD8^+^ T cells aggregate in CRATERs following CpG ODN immune stimulation (A) CRATER coverage in untreated, PBS-, and CpG ODN-injected WT and melanocyte-specific B2M KO, post 4 daily injections. (*n* fish = UT-5, PBS-5, CpG ODN-5, B2M KO CpG ODN-4. Whiskers represent 1.5× the interquartile range. Mann-Whitney U test, *p value = 0.031, ns = 0.42). (B) CD8^+^ T cell density in CRATERs vs. non-CRATER tumor surface in WT and B2M melanocyte-specific KO tumors, post 4 CpG ODN daily injections (*n* fish = WT-5, B2M KO-3. Mean ± SE. Mann-Whitney U test, **p value = 0.007). (C) 3D imaging of melanoma tumors in Tg(cd8α: EGFP) fish after four daily intratumoral injections of PBS or CpG ODN, 24 h post last injection, injected into either B2M WT or KO tumors. Arrows: CRATERs. (D) Percents of ifng mRNA expressing cd8a^+^ cells among total cd8^+^ cells within CRATERs or tumor. (*n* = CpG ODN-4, PBS-3. Mean ± SE. *t* test. *p value = 0.004). (E) RNAscope staining for mCherry, cd8a, and ifng mRNA following four daily injections of PBS or CpG ODN. Dashed white line: tumor surface. Yellow dashed line: CRATER. Arrows: CD8a^+^ cells. (F) infg mRNA expression detected by RNAscope in cells in CRATERs, tumor border (0–50 um from tumor surface) and within the tumor (*n* fish = CpG ODN-4, PBS-3. Mean ± SE. **p value = 0.002, *p value = 0.018). See also Figure S3.

CRATER presence did not restrict tumor growth in untreated zebrafish. However, CRATERs may facilitate CD8^+^ T cell interactions with tumor cells during treatment, leading to tumor killing. The identification of homologous PD-1, PD-L1, and CTLA-4 genes in zebrafish is still ongoing,^25,26^ making ICB treatment in zebrafish challenging. However, CpG ODN, a Toll-like receptor (TLR) agonist in clinical trials for melanoma as an immunotherapy,^27^ elicits inflammation in zebrafish.^28^ We treated tumor-bearing zebrafish with daily intratumoral injections of CpG ODN and monitored them for 24 h after each injection (Figure S3D). Seven daily injections of CpG ODN, but not PBS, arrested tumor growth or shrank tumors (Figures S3E and S3F). Following four daily injections of CpG ODN, but not PBS, tumors presented a statistically significant increase in CRATER coverage (Figure 3A) and large CRATERs (Figure 3C). CD8^+^ T cells maintained an affinity to CRATERs, presenting increased density within CRATERs (Figure 3B) and an increased CRATER/tumor-embedded ratio of CD8^+^ T cell density (Figure S3G). Live imaging showed multiple CD8^+^ T cell-melanoma interactions within CRATERs (Video S3). We visualized disruption of the collagen layer surrounding the tumor mass (Figure S3K), consistent with active T cell infiltration.^29^ We found these effects to be B2M mediated. CpG ODN injections for 7 days did not induce shrinkage of melanocyte-specific B2M knockout (KO) tumors (Figure S3E, lower). Four daily injections of CpG ODN resulted in CD8^+^ T cell recruitment to the tumors site (Figure S3H). However, unlike WT tumors, CD8^+^ T cells remained spatially localized >5 μm away from the tumor or embedded in the tumor parenchyma (Figures S3I and S3J), resulting in reduced CD8^+^ T cell density (Figure 3B) and affinity (Figure S3G) to CRATERs. B2M KO tumors presented no change in CRATER size (Figure 3C, right) or coverage following treatment compared with PBS-injected or untreated WT tumors (Figure 3A). The above indicates that B2M mediates the localization of infiltrating CD8^+^ T cells to CRATERs.

We found that activated CD8^+^ T cells aggregate in CRATERs upon treatment. Interferon gamma (IFN-γ) is expressed by activated CD8^+^ T cell.^30^ Using RNAscope, we found a statistically significant increase in the percentage of *ifng*^+^ CD8^+^ T cells in CRATERs following CpG ODN treatment, compared with elsewhere in the tumor or PBS-injected tumors (Figures 3D and 3E). Moreover, we found that overall *ifng* expression levels were highest within CRATERs following four CpG ODN injections (Figure 3F). This indicates that CRATERs harbor locally elevated levels of IFN-γ, an immune-stimulatory cytokine known to stimulate antigen-presentation expression. Altogether, results suggest that CRATERs may serve as primary sites of the anti-tumor immune response.

### CRATERs are sites of tumor killing following immune stimulation

**Figure 4.**
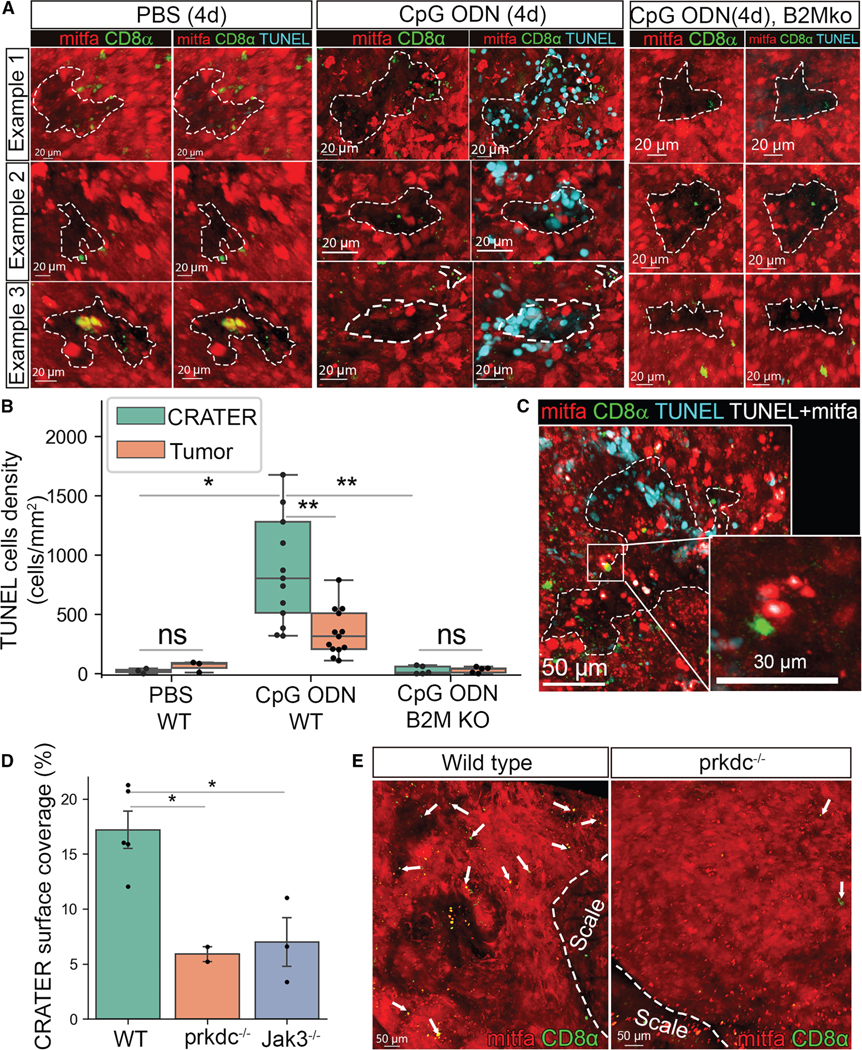
CRATERs are sites of tumor killing and are remodeled by T cells (A) Three examples of CRATERs in 3D confocal images of whole-mount TUNEL assay, in PBS- or CPG ODN-injected tumors (four times daily), WT or melanocyte-specific B2M KO. (B) TUNEL^+^ cell density in CRATERs and non-CRATER tumor area. (Image quantification. *n* = PBS WT-3 fields of view [FOV] from 3 fish, CpG ODN WT-12 FOV from 8 fish, CpG ODN B2M KO-5 FOV from 2 fish. Whiskers represent 1.5× the interquartile range. *t* test. *p* values: *PBS vs. CpG ODN WT = 0.03, **CPG ODN: CRATER vs. tumor = 0.005, **CpG ODN: WT vs. B2M KO = 0.007). (C) 3D imaging of whole-mount TUNEL staining of CpG ODN-treated tumor. White dashed lines: CRATER. Rectangle area enlarged. Image slightly rotated. (D) CRATER coverage in tumors of cd8α:EGFP (WT), cd8α:EGFP;prkdc^−/−^, or jak3^−/−^ transgenic fish (*n* fish = WT-5, prkdc^(−/−)^-2, jak3^(−/−)^-3 fish. Mean ± SE. *t* test, *p* values: *WT/cd8a:EGFP;prkdc^(−/−)^ = 0.011, * WT/jak3^(−/−)^ = 0.01). (E) Representative 3D confocal images of melanoma in WT Tg(cd8a:EGFP) and Tg(cd8a:EGFP;prkdc^(−/−)^) fish. Arrows: CD8^+^ T cells. See also Figure S3.

To assess whether tumor killing occurs within CRATERs, we performed a whole-mount terminal deoxynucleotidyl transferase dUTP nick end labeling (TUNEL) assay, enabling a 3D view of the tumor surface, on CpG ODN-treated vs. PBS- or non-treated tumors and B2M-intact or B2M-depleted tumors. Very few apoptotic TUNEL^+^ cells were found on the surface of PBS-treated tumors or CpG ODN-treated B2M KO tumor after four daily injections (Figure 4A). CpG ODN-injected WT tumors, however, presented multiple TUNEL^+^ cells preferentially within CRATERs (Figure 4A). Image quantification revealed that TUNEL^+^ cell density was significantly higher in CRATERs compared with elsewhere in the tumor following CpG ODN treatment of WT, but not B2M KO, tumors (Figure 4B). Moreover, CD8^+^ T cells could be found in direct contact with TUNEL^+^ melanoma cells (Figure 4C), an association consistent with T cell-mediated cytotoxicity. Taken together, these results indicate that cell death and tumor killing primarily occurred within CRATERs upon treatment. These results further indicate that CD8^+^ T cells contribute to sculpting CRATER architecture. We compared WT tumors to tumors from two distinct T cell-depleted zebrafish lines: Tg(cd8α:EGFP;prkdc^(−/−)^) zebrafish lacking T cells and B cells^11^ and jak3^(−/−)^ transgenic zebrafish lacking T cells and putative natural killer (NK) cells.^11^ CRATER coverage in tumors of both T cell-deficient strains was significantly lower, but not absent, compared with tumors of WT fish (Figures 4D and 4E), indicating that T cells contribute to CRATER coverage in tumors. The rare CRATERs existing in prkdc^(−/−)^ zebrafish tumors exhibited *b2m* mRNA expression similar to WT, as noted by RNAscope (Figure S3L), suggesting that *b2m* expression levels in the melanoma cells were not affected by the low levels of CD8^+^ T cells.

Transforming growth factor β (TGF-β) inhibitors (TGF-βi) are currently in clinical trials as immunotherapies.^31^ TGF-β inhibition for 24 h by SB431542 also led to an accumulation of CD8^+^ cells in CRATERs (Figures S3M and S3N). Using long-term time-lapse imaging of TGF-βi-treated tumors, we saw a cluster of CD8^+^ T cells form lengthy interactions with an mCherry^+^ melanoma cell at the scale edge. In this movie (Video S4), after ∼14 h, the CD8^+^ cell cluster transferred the mCherry^+^ fragment to a large, mpeg^+^/CD8^+^ cell, which then retained a piece of the mCherry fragment on its dendrites (Figures S3O and S3P). Though the final confirmation of the CD8^+^/mpeg^+^ cells as DCs still warrants further investigation, this imaging may be suggestive of the initial steps of antigen uptake, captured live in this movie. We further noticed that, 24 h post CpG ODN injection, cd8a:EGFP^+^ cells with dendrites carried mCherry fragments along the tumor border from the tumor peak to the scale edge (Video S5). This is consistent with previously reported antigen mobilization by CD8^+^ DCs,^32^ but final confirmation of the CD8^+^ cells’ identity is still warranted.

Last, the murine model of transplanted melanoma is widely used in cancer immunology studies. OVA-transfected melanoma cell line transplantation, followed by transfer of OVA-sensitive T cells (OT), enables evaluation of the effect of antigen recognition by CD8^+^ T cells on tumor eradication but yields a rapid expansion of exogenous tumor mass with very high antigen content. We found in this model that focal attrition of the tumor surface, similar to CRATERs, occurs only when CD8^+^ T cells are transferred, as seen by 3D confocal imaging of tumors (Figure S4B). OT cells transfer resulted in widespread attrition of the tumor surface (Figures S4A and S4B), indicating a potent anti-tumor immune response that excided the zebrafish model. B2M expression extended beyond the areas of tumor attrition, and CD8^+^ T cells localized with it, nonspecific to tumor attrition sites (Figures S4C and S4D). In this model, a potent antigen, OVA, is strongly expressed by all the tumor cells transplanted into mice, rendering the tumor surface visible to the transferred CD8^+^ T cells. Differential antigen presentation could potentially occur as the tumor develops within the animal. However, these results indicate that such a process is more pronounced in tumors developing endogenously, as in the zebrafish model, and in human melanoma, yielding the CRATERs presented above. Thus, we next examined whether structures similar to CRATERs are found in human melanoma tumors.

### Human melanoma tumors exhibit CRATERs harboring densely packed CD8^+^ T cells

**Figure 5.**
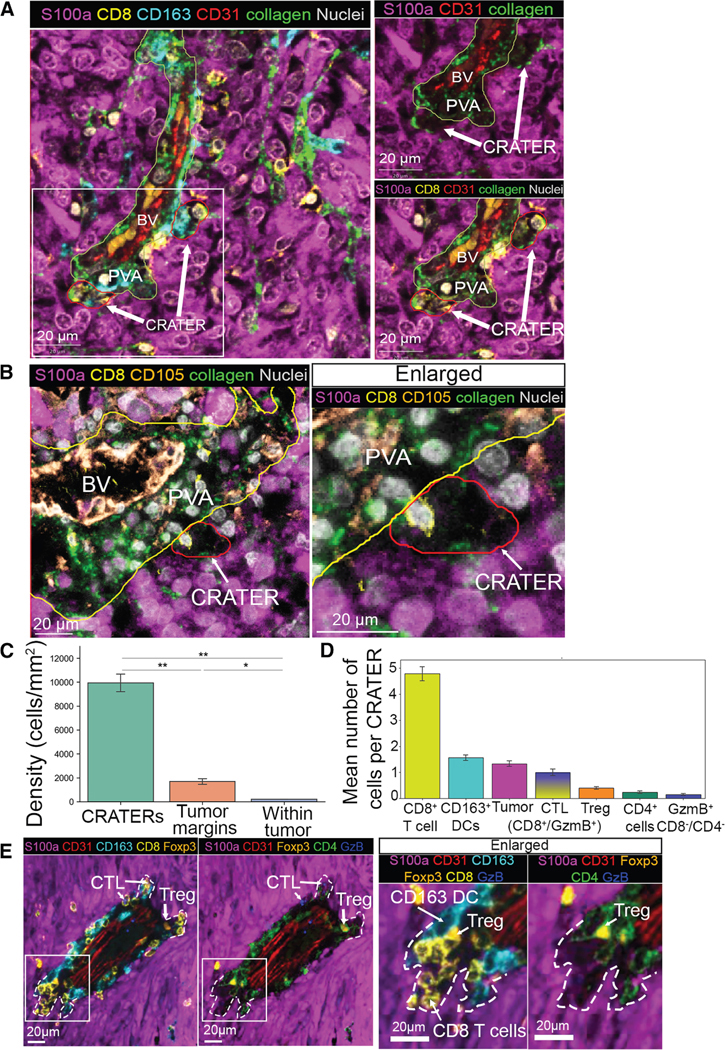
CRATERs are found in human melanoma, extending from the tumor margins (A) Left: mIF image of a (PVA) within a cutaneous melanoma tumor. Red lines: CRATERs. Yellow line: PMB. Right: rectangle area enlarged. Upper image: collagen fibers lining the CRATERs. Lower image: CD8^+^ T cells within CRATERs. (B) CD105 expression in perivascular area (yellow line) but not in the CRATER (red line). Representative image of 13 melanoma samples. (C) CD8^+^ T cell density in CRATERs, tumor margins (PMB), and embedded in the S100a^+^ tumor mass (within tumor). (*n* = 2 tumors, 714 CRATERs. Mean ± SE, *t* test, *p* values: **CRATER/border = 0.008, **CRATER/tumor = 0.005, *0.023). (D) Mean number of cells per CRATER for each cell type (*n* = 714 CRATERs, mean ± SE). (E) Representative CyCIF image showing location of CD8^+^ T cells, CD163^+^ DCs, CD4^+^ cells, CD4^+^/FOXP3^+^ T regulatory cells, and CD8^+^/GzmB^+^ cytotoxic T cells at the perivascular and CRATER areas. Abbreviations: BV, blood vessel; PVA, perivascular area; PMB, perivascular-melanocytes boundary; CTL, cytotoxic T cells; Treg, T regulatory cells; GzmB, granzyme B; DCs, dendritic cells (CD11c^+^). See also Figures S5 and S6.

To determine whether structures akin to CRATERs exist in human melanoma, we quantitatively analyzed a total of 32 human melanoma samples (of 29 cutaneous and 4 mucosal melanoma), including 7 primary melanoma samples and 25 metastases, using cyclic immunofluorescence (CyCIF, 2 samples) and multiplexed immunofluorescence (mIF) (30 samples, see Table S1). We found that both primary melanoma and melanoma metastases, cutaneous and mucosal, contained CRATERs. The CRATERs are small, ∼20-μm pockets (Figure S5A) breaching into the melanoma cell mass from the stromal layers of the tumor, both at the tumor border with the adjacent tissue (Figure S5B) and at the boundaries of the melanocytic compartment with the perivascular area, which is the stromal layer that encircles blood vessels (i.e., perivascular-melanocytic boundaries [PMB]) (Figures 5A and S5C). These stromal regions have been shown to contain DCs and T cells in mice^33^ and human.^34^ CRATERs were lined by melanoma cells throughout—sides and bottom—creating an irregularly shaped pocket (Figures 5A, 5B, S5B–S5F, S5L, and S5N). As in zebrafish, one or two collagen fibers extended into the CRATERs from the collagen layer of the perivascular area (Figures 5A and S5D–S5F), distinguishing the CRATERs from it. We used tumor and CRATER segmentation (Figure S5G), and cell segmentation (Figure S5H) followed by cell clustering of CyCIF data (Figure S5I), to calculate cell density and define the CRATER cellular composition. CD8^+^ T cell density was highest within CRATERs, compared with either the PMB (tumor margins) or cells embedded within the tumor mass (Figure 5C), indicating that CD8^+^ T cells cluster within CRATERs. Per cellular composition, CRATERs mostly harbor CD8^+^ T cells and CD163^+^/CD11c^+^ DCs, (Figures 5D and 5E). Granzyme B (GzmB)^+^ cytotoxic CD8^+^ T cells could be found in CRATERs in lower numbers than GzmB^−^/CD8^+^ T cells (Figures 5D and 5E). CD4^+^ T cells were mostly found outside the CRATERs (Figures 5D and 5E). CD4^+^/Foxp3^+^ Tregs were found embedded in the tumor mass or in the perivascular area, occasionally close to the CRATER edges but rarely inside (Figures 5D and 5E). GzmB^+^ cells that were not T cells were rarely found within CRATERs (Figures 5D and 5E).

During tumor development, we found indentations similar to CRATERs only in the vertical phase of invasive growth (Figures S6C and S6D) and not in the earlier, radial growth phase of primary melanoma (Figures S6A and S6B). This is consistent with the known prognostic significance of the presence and extent of T cells that infiltrate the tumor-stromal interface of melanomas in the vertical/nodular stage of their growth.^34,35^

### CRATERs in human melanoma are sites of antigen presentation, rich in PD-L1

**Figure 6.**
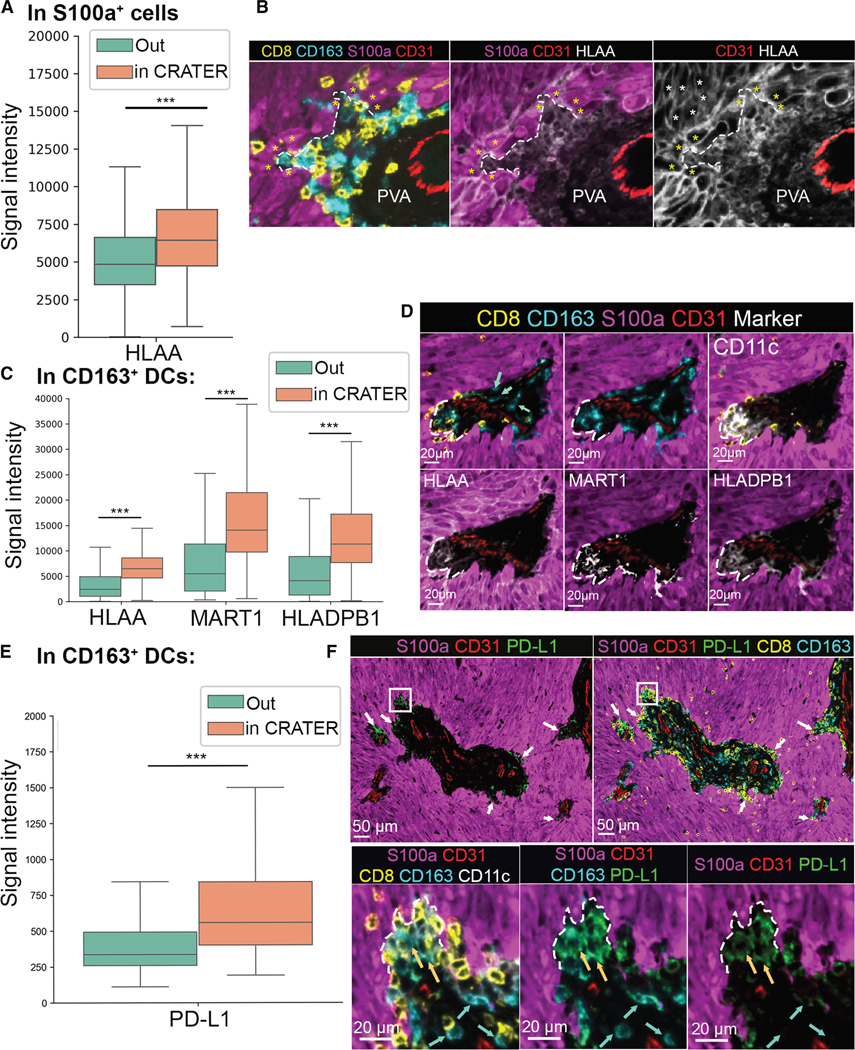
CRATERs in human melanoma are sites of high antigen presentation molecules and PD-L1 expression (A) Signal intensity distribution of HLA-A staining in S100a^+^ melanoma cells in CRATERs compared with elsewhere in the tumor (“Out”) (*n* = 2 tumors, 714 CRATERs. Whiskers represent 1.5× the interquartile range. Mann-Whitney U test. ***p value < 0.001). (B) Representative image presenting HLA-A staining in CRATER (dashed white line). Yellow asterisks: melanoma cells lining the CRATER. White asterisks: melanoma cells located away from the CRATER. (C) Signal intensity distribution of HLA-A, MART-1, and HLA-DPB1 staining in CD163^+^/CD11c^+^ DCs in CRATERs compared with elsewhere in the tumor (“Out”) (*n* = 2 tumors, 714 CRATERs, whiskers represent 1.5× the interquartile range. Mann-Whitney U test, ***p value < 0.001) (D) Representative image of CRATER (white dashed line), stained for CD163^+^ HLA-A, MART-1, and HLADPB1. (E) Signal intensity distribution of PD-L1 staining in CD163^+^/CD11c^+^ DCs in CRATERs compared with elsewhere in the tumor (“Out”) (*n* = 2 tumors, 714 CRATERs. Whiskers represent 1.5× the interquartile range. Mann-Whitney U test, ***p value < 0.001). (F) Upper: low magnification of a tumor area. High PD-L1 staining within CRATERs. White arrows: CRATERs. Lower: rectangle area enlarged, showing a CRATER (dashed white line) containing PD-L1^+^/CD163^+^/CD11c^+^ DCs. Teal arrows: CD163^+^/CD11c^+^ DCs (with low PD-L1 staining) outside the CRATER. Orange arrows: CD163^+^CD11c^+^ (PD-L1^+^) DCs inside the CRATER. All images and quantifications are CyCIF data.

In zebrafish, *b2m*, encoding a key component of MHC class I, was highly expressed in CRATERs. In human melanoma, we found increased expression of human leukocyte antigen A (HLA-A), corresponding to MHC class I, in the S100a^+^ tumor cells lining the CRATERs (Figures 6A and 6B). Moreover, CD163^+^/CD11c^+^ DCs within CRATERs presented a statistically significant increase in HLA-A and HLA-DPB1 levels, corresponding to MHC class II, and MART-1 melanoma antigen, compared with elsewhere in the tumor (Figures 6C and 6D). This suggests that DCs within CRATERs contribute to antigen presentation within human CRATERs. PD-L1 expression was significantly higher in CD163^+^/CD11c^+^ DCs within the CRATERs compared with the rest of the tumor (Figures 6E and 6F), consistent with previous reports of PD-L1 expression by DCs and macrophages at the melanoma margins.^34^ Of note, PD-L1 staining was higher in CD163^+^ DCs contacting the CD8^+^ T cells in the CRATERs compared with CD163^+^ cells at the same perivascular area (Figure 6F), suggesting that CD8^+^ T cells are also exposed to PD-L1 within the CRATER. CD8^+^ T cells within and outside of the CRATERs expressed markers of T cell dysfunction (Figure S5N), suggesting that CD8^+^ T cell dysfunction is not determined in the CRATER.

### CRATER density is correlated with response to ICB therapy

**Figure 7.**
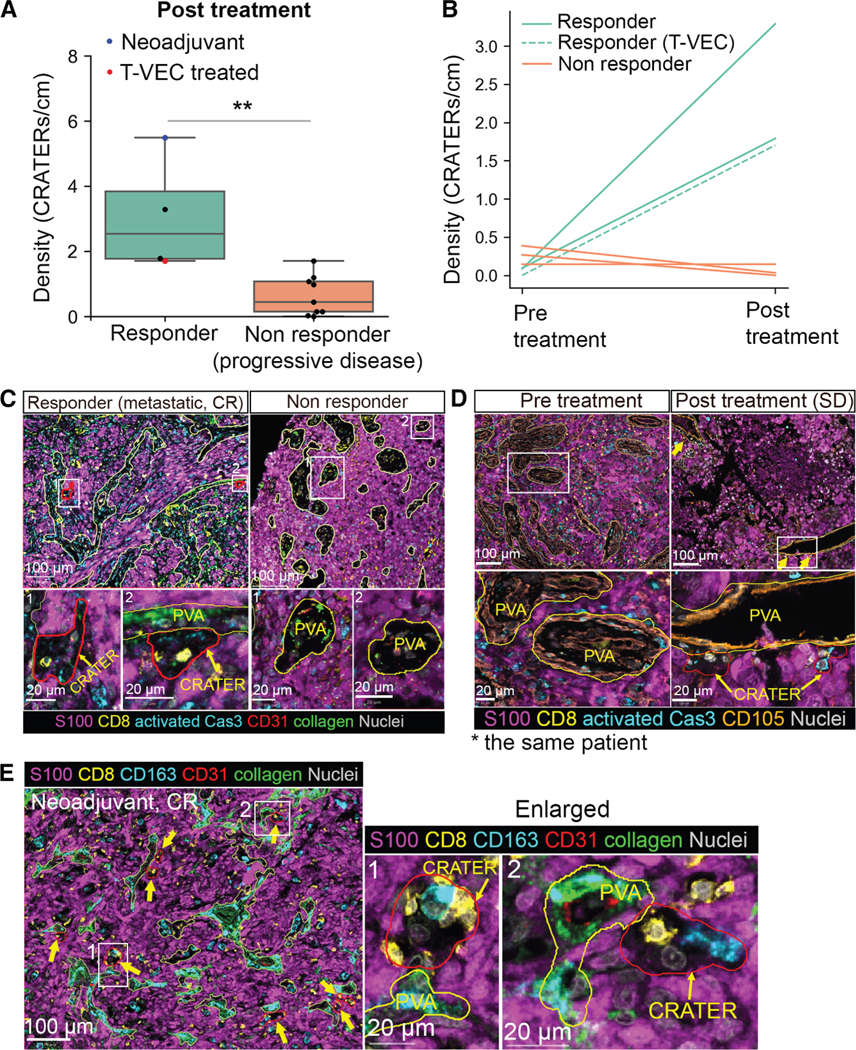
CRATER density is increased following successful ICB treatment (A) CRATER linear density (number per cm PMB) in post ICB treatment patients’ samples (*n* = 4 responders [SD, CR], 9 non-responders [PD]. Whiskers represent 1.5× the interquartile range. *t* test. *p value = 0.0027). Black dots: metastatic setting. Red dot: T-VEC. Blue dot: neoadjuvant setting. (B) CRATER linear density in pre- and posttreatment samples from the same patient, showing altogether three responders and three non-responders. Each line represents a patient. Dashed line: T-VEC treated. (C) Representative images of mIF-stained samples from a responder and a non-responder. Top: low-magnification view of the sample. Bottom: rectangles enlarged. Yellow lines: PMB. Red lines: CRATERs. (D) mIF for melanoma, CD8, apoptotic cells (activated cas3), and CD105 of biopsies taken before and after treatment from the same patient, who presented SD post treatment (#13 in Table S1). Top images: low-magnification view. Bottom images: rectangles in the respective top image, enlarged. (E) mIF image of biopsy taken during neoadjuvant treatment that presented CR. Left: low-magnification image. Yellow arrows: CRATERs. Right: rectangle areas, enlarged. See also Figure S7.

CRATERs can be pathologically identified by their location at the borders of stromal and melanocytic compartments, either at the tumor border or at the PMB. CRATER identification does not require the presence of CD8^+^ T cells. CRATERs are 20–50 μm in size (Figure S5A) and form an irregularly shaped gap of staining in the tumor that is surrounded by melanoma cells and has few nuclei. Hematoxylin and eosin (H&E) staining is not sufficient to identify CRATERs, as the gap is not clearly visible (Figure S5J). CRATERs contain only one or two collagen fibers. There are no blood vessels, CD105^+^ stromal and endothelial cells (Figures 5A, 5B, S5K–S5M), or aSMA^+^ pericytes (Figures S5B and S5C) in CRATERs. Additional markers include elevated expression of HLA-A, MART-1, and PD-L1, which highlight the CRATERs by immunostaining (Figures 6D and 6F). CRATERs harbor one or two CD163^+^ DCs and do not harbor CD4^+^ T cells (including Tregs). All the above will enable a clear identification of a CRATER. In zebrafish, CRATER coverage increased during therapy that hampered tumor growth. Hence, we examined whether the extent of CRATERs created during treatment correlates with the outcome of that treatment in patients. We examined pathological samples of metastases extracted after a patient has been given ICB treatment. We calculated the number of CRATERs per PMB length, i.e., CRATER linear density, which should indicate the extent of response taking place, and correlated it with the actual clinical outcome of the given treatment. Samples from patients who were clinically responsive to treatment are rare, as the patients may not require additional surgery. Nevertheless, we were able to compare four cases of patients who presented a durable clinical benefit, i.e., a sustained best overall response of stable disease (SD), partial response (PR), or complete response (CR), termed here “responders,” with nine patients who presented a PD, termed here “non-responders,” all per RECIST v.1.1 for at least two restaging scans (Table S1). We found that biopsies collected post treatment from patients who became responders presented a significantly higher CRATER density than those from patients who became non-responders (Figures 7A and 7C). Analysis of samples that were taken post ICB treatment given in the metastatic setting may pose a limitation because the resected metastases have, in fact, presented some resistance to treatment and thus had to be removed (Figure 7A, black dots). However, the increase in CRATER density suggests that the anti-tumor immune response did occur within those resected tumors but not in metastases removed following unsuccessful treatment. This highlights the potential of CRATER measurement to indicate an ongoing immune response. In the neoadjuvant setting, treatment is given prior to surgical removal of the residual tumor. In this setting, such biopsies do not pose the above limitation, as a selection for resistant metastases does not occur. We obtained one biopsy taken post neoadjuvant ICB treatment. It presented the highest CRATER density along the PMB (Figures 7A [blue dot] and 7E), suggesting that high CRATER density is even more pronounced in samples that are not selected for resistant tumors. In zebrafish, we have used CpG ODN, which mimics a viral response, as an immune stimulus. For patients, CpG ODN is still in clinical trials. However, talimogene laherparepvec (T-VEC) is a clinically approved modified herpes simplex virus that attacks and lyses melanoma cells and, though not identical to CpG ODN, elicits a broad immune response.^36^ Among the posttreatment cohort, one patient presented a CR 5 months after T-VEC treatment completion given after an unsuccessful round of ICB treatment (Table S1, patient #12). The biopsy analyzed was taken at the end of the T-VEC treatment and presented high CRATER density (Figure 7A, red dot), supporting our zebrafish findings. Moreover, we obtained a 3D reconstruction of human melanoma using reflectance confocal microscopy (RCM) imaging, performed in patients *in vivo*.^37^ RCM in untreated tumors exhibited CRATER-like structures resembling zebrafish CRATERs, harboring lymphocyte-like cells (Figure S7A; Video S6). RCM on a patient who underwent T-VEC treatment displayed enlarged CRATER-like areas that harbored multiple lymphocyte-like cells (Figure S7A), demonstrating lymphocyte accumulation within enlarged CRATERs following T-VEC. Altogether, we found that successful treatment was accompanied by CRATER abundance, suggesting that high CRATER density reflects an effective anti-tumor immune response.

We next examined the biopsies taken before treatment (i.e., pre-treatment samples) from six patients among the posttreatment cohort presented above and compared CRATER density before and after treatment in the same patient. We found that ICB treatment led to an increase in CRATER density in responding patients but not following unsuccessful treatment (Figures 7B and 7D). We noted that, in samples of non-responders that presented CD8^+^ T cell infiltration, CD8^+^ T cells were embedded diffusely in the S100a^+^ tumor mass or entered the tumor as single cells or doublets, rather than clustering within CRATERs (Figures S7B–S7E), altogether suggesting that CRATERs may represent a visual sign of an effective immune response in tumors. We further noted that CRATERs can be found clustered regionally within the whole tumor mass, which may indicate areas of immune response (Figure S7F). However, more extensive prospective clinical trials are needed to establish the power of CRATERs as an indicator of a region of effective immune response. Analysis of CRATERs in pre-treatment samples did not correlate with treatment outcomes (Figure S7G). Nevertheless, CRATER presence in the posttreatment biopsy was statistically associated with clinical outcome.

We found that 73% of CRATERs contained apoptotic, activated Cas3^+^ cells, either contacting CD8^+^ T cells or located away from them (Figures 7C, 7D, and S7H). We detected an increase in the percentage of CRATERs harboring direct interactions between CD8^+^ T cells and apoptotic cells upon successful treatment (Figure S7I), indicating that, despite the high levels of PD-L1 within CRATERs, CD8^+^ T cells engage with apoptotic melanoma cells within CRATERs upon successful treatment.

Last, we reviewed fifteen samples of non-small cell lung cancer (NSCLC) at different stages of disease for CRATERs. We noted clusters of CD8^+^ T cells in PD-L1-high pockets breaching into the cytokeratin^+^ tumor mass in large-volume tumors (Figure S7J, images 3,4) but not in earlier stages (Figure S7J, images 1,2). These pockets were 20–50 μm in diameter, contained CD8^+^ T cells and CD163^+^ cells, and lacked dense collagen fibers and CD31, similar to CRATERs found in melanoma tumors (Figures S7K and S7L). This indicates that CRATER formation is not restricted to melanoma but may be a general feature of an effective immune response in solid tumors and should be examined in detail.

## DISCUSSION

Melanoma is susceptible to immunotherapy, which may abrogate even metastatic disease. Study of the spatial and functional dynamics of immune cell interactions with melanoma cells within tumors has been limited by current imaging techniques.^38^ Using zebrafish, we were able to combine confocal 3D imaging of endogenous melanoma tumors with extended time-lapse imaging in live, anesthetized, and intact animals and record immune-tumor interactions for over 15 h. Zebrafish present a highly conserved immune system,^8^ but differ in some respects. The major pathways directing ICB, such as the PD-1/PD-L1 axis^24^ and CTLA-4,^25,26^ have not been fully annotated in zebrafish. Despite these differences, a conserved concept emerged of spatially distinct sites with elevated antigen presentation that facilitate CD8^+^ T cells-tumor cells interactions. We termed these sites CRATERs.

Following immune stimulation, the CRATERs expand and facilitate an effective immune response against the tumor, harboring IFN-γ^+^, activated CD8^+^ T cells interacting with apoptotic cells in zebrafish. The zebrafish could not provide a platform to directly examine ICB treatment effect on CRATERs remodeling. However, we did find a consistent elevation in CRATER density in patients responding to ICB treatment compared with non-responders, even when T cell infiltration occurred. This suggests that CRATER density at the tumor margin can indicate an efficient anti-tumor immune response taking place following ICB therapy. To date, efficacy of therapeutic response to immunotherapy is assessed mainly by estimating the degree of tumor necrosis and fibrosis.^39^ Indicators of CD8^+^ T cell infiltration have been associated with patient survival and treatment outcomes, but direct evidence of effective immune cell-tumor cell interaction is still missing. The presence and location of tumor-infiltrating lymphocytes (TILs) have been linked to patient survival.^3^ Recently, the presence of tertiary lymphoid structures (TLS), which form near tumors post treatment as an organized lymph-node-like structure, were also correlated with improved survival post treatment.^4^ TLS were linked with CD8^+^ T cell infiltration into tumors^40^ and were suggested to facilitate antigen presentation as well as T cell expansion and activation.^41^ The presence of TLS and brisk TIL infiltration indicates that activated CD8^+^ T cells enter the tumor. CRATERs denote the next step, indicating that this infiltration indeed yields an effective tumor killing that controls tumor growth. Pending thorough clinical verification, CRATER linear density measurement can further refine the pathological assessment of treatment response. It may serve, together with TLS and TIL measurement, to more accurately assess the efficacy of an ongoing treatment and improve treatment outcomes.

We noted IFN-γ gene expression preferentially within CRATERs following CPG ODN treatment in zebrafish. IFN-γ enhances T cell cytotoxicity as well as MHC class I and PD-L1 expression,^42–44^ and both are increased in CRATERs. Local inflammation and IFN-γ secretion may have a function in establishing CRATER characteristics, as seen in this study. However, IFN-γ staining is highly challenging due to its diffusive nature, making it difficult to fully confirm this notion. T cells themselves may shape tumor architecture through apoptosis and expand CRATER formation, as lack of T cells resulted in low CRATER coverage.

Increased antigen recognition within CRATERs underlies CD8^+^ T cell localization to CRATERs. B2M loss was previously associated with a poor response to ICB treatment^45^ and is required for an effective CD8^+^ T cell-mediated anti-tumor immune response.^46^ However, there are patients lacking B2M that respond to ICB therapy.^47^ In such instances, reproduced in mice, CD8^+^ T cells did not efficiently attack the tumor, but the lack of B2M enabled the activation of NK cells and CD4^+^ T cells that affected tumor growth.^47^ As we found that CD4^+^ T cells and NK cells are likely not found in CRATERs in human melanoma, CRATERs indicate a CD8^+^ T cell-dependent anti-tumor immune response. It is yet to be explored which mechanisms propagate the anti-tumor immune response by other arms of the immune system. Our murine study suggests that localized antigen presentation and CRATER formation may be attributed to the gradual development of the tumor. Murine models are instrumental in cancer immunity and, unlike in zebrafish, ICB treatment is available in mice. Our results suggest that murine models of endogenous tumor formation, without artificial antigen presentation, should be characterized for CRATER formation to further explore the mechanisms regulating CRATER formation during ICB treatments.

Moreover, we identified structures similar to CRATERs in human lung cancer, indicating that CRATERs are likely not limited to melanoma and may form in other solid tumors.

Improved understanding of how CRATERs may be leveraged to effect a more robust anti-tumor immune response may ultimately increase immunotherapy efficiency. Moreover, robust clinical trials should follow to assess CRATERs as an early pathological marker for ICB treatment efficacy, which is currently not available in the clinic. This will serve to optimize clinical treatment regimens to enhance ICB success and patient well-being.

### Limitations of the study

Robust clinical studies of multiple patients, preferentially in the neoadjuvant setting, are needed to establish CRATERs’ potential as a biomarker for ICB success. However, this was not the standard of care when the samples used in these studies were obtained. CRATERs can best be seen when a mass of malignant cells is present, making their use challenging for single-cell dissemination or desmoplastic melanoma. Though the focus of our study is CD8^+^ T cells, DCs emerged as participants in CRATERs. The role of DCs, including CD8^+^ DCs and CD163^+^ DCs, in CRATERs was not explored. Further studies, including a detailed characterization of DCs in zebrafish, are warranted.

### RESOURCE AVAILABILITY

#### Lead contact

Further information and requests for resources and reagents should be directed to, and will be fulfilled by, the lead contact, Leonard I. Zon (leonard.zon@enders.tch.harvard.edu).

#### Materials availability

Reagents, plasmids, and zebrafish lines generated in this study will be made available on request.

#### Data and code availability

- RNA-seq and ATAC-seq zebrafish data are available at NCBI Gene Expression Omnibus (GEO) under the accession number GEO: GSE213286.
- Code for identification of tumor attrition in murine 3D samples is available on GitHub: https://github.com/AsafTl/3D-Polynomial-Surface-Fitter.
- Any additional information required to reanalyze the data reported in this paper is available from the lead contact upon request.

## STAR★METHODS

### METHOD DETAILS

#### Zebrafish husbandry

Zebrafish (*Danio rerio*) were bred and maintained in accordance with Animal Research Guidelines of the Institutional Animal Care and Use Committee at Harvard University (protocol #11–21), following standard protocols (at 28.5°C water temperature and a 14/10-hour light/dark cycle). Individual mating of the transgenic fish- Tg(*cd8α*:EGFP) and Tg(*lck*:mCherry) (generation described below), Tg(*flk*: EGFP),^65^ Tg(*fli1ep*:dsred),^66^ or Tg(BRAF^V600E^/p53^null^/nacre^null^)^5^ were done to create double transgenic zebrafish lines. Jak3^P369fs^ heterozygous zebrafish (kindly provided by David Langenau) and prkdc^D3612fs^ were in-crossed, injected with tumor constructs using the MAZERATI approach (described below), and genotyped as previously described.^16^ All Jak3^P369fs^ zebrafish were raised on a standalone rack with an antibiotic mix added weekly (dosage per liter fish water): 25mg Penicillin G Sodium (Sigma Aldrich, P3032), 36mg Streptomycin Sulfate (Sigma Aldrich, S6501), 0.1mg Amphothericin B (Santa Cruz Biotechnology, SC-202462B), 8mg Keflex/Cefalexin (Fish Mox Fish Flex), 65uL Prazipro/Praziquantel (Hikari Usa, AHK73254).

#### Generation of *cd8a*:EGFP transgenic zebrafish

A 3822 base pair sequence upstream of the zebrafish CD8a gene, that contains open chromatin regions identified in ATAC-Seq of lck: GFP^+^ cells, was cloned from genomic DNA of zebrafish obtained from adult zebrafish of AB strain, in two stages. First, a sequence containing the promoter was cloned using the following primer sequences: FW: 5’-CTGTATCTCTGTGTGTGCGT-3’ Rv: 5’-GTGGCACCCTTTAACATGATCG-3’. The product of this PCR amplification was subsequently used as a template to clone the specific promoter site, using the following primer sequences: FW: 5’- TGAGGTTCGTCAGCAGACTTG-3’ Rv: 5’-CCAGTTTCCAGCACAAGCATTC-3’. The fragment was incorporated into a pENTR 5’ TOPO TA vector and was used to drive EGFP expression using the multisite Gateway cloning technology, with the pDestTol2CG2 as a backbone plasmid, which induces EGFP expression in the heart. This facilitated selection of zebrafish in which the plasmid integrated into their genome. Single-cell embryos of casper background zebrafish^67^ were injected with the plasmid and Tol2 mRNA at 1:1 ratio. Transgenic founders were selected based on EGFP fluorescence in the heart. Fish were bred for three generations to create stable transgenic zebrafish.

#### Generation of *lck*:mCherry and (*cd8α*:EGFP;*lck*:mCherry) transgenic zebrafish

Lck reporting transgenic zebrafish were produced by using the 5.5-kb sequence upstream of the *lck* start codon (kindly provided by David Langenau)^16^ to drive mCherry expression, using the multisite Gateway cloning technology.^68^ The vector was generated using the pDestTol2pA2 backbone vector. Single-cell embryos of Tg(cd8α:EGFP) zebrafish or of casper^67^ zebrafish were injected with the plasmid and Tol2 mRNA at 1:1 ratio, to create double transgenic or single lck:mCherry transgenic fish respectively. Transgenic founders were selected based on mCherry fluorescence in the thymus.

#### Alexa Flour 594 conjugate Ovalbumin uptake assay

Ovalbumin Alexa Flour 594 conjugate (Invitrogen) was dissolved in PBS to a 5mg/ml stockconcentration. 5ug OVA-AF594 was injected intra peritoneal at a volume of 4ul to anesthetized

Tg(CD8α:GFP) zebrafish weighing ∼880mg. 24 hours post injection, the kidney marrow washarvested from either OVA-AF594 injected or PBS injected zebrafish, analyzed and sortedusing FACs Aria III (Becton Dickinson), as described above, to be used for inverted confocalimaging, or the zebrafish thymus was imaged by confocal in vivo imaging as described above.

#### Generation of melanoma tumors

Melanoma tumors were generated using two approaches. The first approach relies on re-introducing the ability to form melanocyte to a transgenic zebrafish already harboring the human the human BRAF(V600E) mutation and lack the p53 gene and mitfa gene.^7^ The melanocytes of this fish will harbor the mutations and a subset of them will develop into melanoma tumor. In detail, we used injection of a MiniCoopR vector^7^ carrying an mitfa mini-gene and mCherry expressed under the mitfa promoter, together with Tol2 mRNA at 1:1 ratio, into a single cell-stage embryo of Tg(*cd8α*:EGFP;BRAF^V600E^;p53^null^;nacre^null^) zebrafish, created by breeding of the newly generated cd8a:EGFP line described above with the established (BRAF^V600E^;p53^null^;nacre^null^) line, followed by genotyping to identify germline presence of BRAF^V600E^ and the p53^null^;nacre^null^ mutation in the newly created line, as previously described,^7^ and propagate the fish line as a stable line. This model was used throughout this study except for the figures detailed below for the following second model. The second model was used to create melanoma in zebrafish lines that do not harbor the BRAF(V600E) and lack p53, such as transgenic zebrafish reporting blood vessels using the MAZERATI system.^24^ In this model, the BRAF(V600E) was introduced de novo and the zebrafish p53 gene was mutated using a tissue specific CRISPR, as described in Ablain et al.,^24^ in the single cell stage embryo. This produced zebrafish in which a portion of their melanocytes harbored the above-described mutations that yield melanoma tumors. In detail, single cell-stage embryos were injected with a cocktail of three vectors: a MiniCoopR vector expressing an mitfa mini-gene and BRAF^V600E^, a CRISPR MiniCoopR vector expressing an mitfa mini-gene, mitfa-driven Cas9, a gRNA targeting Tyrosinase (GGACTGGAGGACTTCTGGGG), and a gRNA targeting p53 (GGTGGGAGAGTGGATGGCTG), and a vector expressing BFP or mCherry under the mitfa promoter. This model was used in Figures 1A, 1F–1H, and S2F–S2J. We did not find a significant difference in T cell behavior and distribution between these two approaches, and our findings were corroborated in tumors generated by either approach.

#### Tissue harvesting, flow cytometry and sorting

Zebrafish were euthanized in ice water. Thymus, spleen and kidney marrow or melanoma tumors were harvested under fluorescent stereo microscope Leica M165 FC. The tissues were meshed into a single cell suspension in HBSS medium (Gibco) enriched with 0.2% Fetal calf serum (FCS, Atlanta Biologicals), using a pestle. The samples were then filtered through a 40 μm filter, centrifuged at 470g for 5 minutes. The supernatant was removed, and the samples were resuspended each in 400μl HBSS+0.2% FCS. Samples were analyzed and cells were sorted into 2ml of HBSS+0.2%FCS on a FACs Aria III (Becton Dickinson) with a 100 μm nozzle at 22psi, using the FACSDiva software (Becton Dickinson).

#### RNA collection and extraction

Fluorescent tagged cells sorted to purity of over 90% were used for RNA sequencing. The cells were centrifuged at 470g for 5 minutes, supernatant was removed and the cells were re suspended in 500 μl Trizol (Invitrogen) and kept in −20°C until RNA extraction. RNA was extracted using small scale RNA isolation protocol. Briefly, samples were thawed to RT and chloroform was added at 1:5 ratio. Samples were then centrifuged at 12,000g for 15 minutes at 4°C and the aqueous phase containing the RNA was collected and isopropanol was added. Samples were kept over night at −20°C and were centrifuged at 12,000g for 10 minutes at 4°C to collect RNA. The RNA pellet was washed 3 times with 75% ethanol in nuclease-free water, re-suspended in 10 μl nuclease-free water and kept in −80°C until use.

#### Ultra low input RNA sequencing (bulk RNA-seq)

CD8α:EGFP^+^ cells were sorted from thymi of 7 weeks old (juvenile) CD8α:EGFP transgenic zebrafish or from melanoma from melanoma bearing Tg(*cd8α*:EGFP;BRAF^V600E^;p53^null^;nacre^null^) zebrafish. 1 ng of RNA was used from each sample. cDNA was prepared using SMART-Seq^®^ Ultra^™^ low Input RNA kit for sequencing (Clontech) according to the manufacturer’s instructions. Libraries were prepared using Nextera XT DNA library preparation kit (Illumina) and Nextera XT index kit (Illumina) according to the manufacturer’s instructions. Libraries prepared went through quality control analysis using an Agilent Bioanalyzer. Samples with appropriate nucleosomal laddering profiles were selected for next generation sequencing using Illumina Hiseq 2500 platform.

#### RNA-seq analysis

Quality control of RNA-Seq datasets was performed by FastQC (http://www.bioinformatics.babraham.ac.uk/projects/fastqc/) and Cutadap^52^ to remove adaptor sequences and low-quality regions. The high-quality reads were aligned to UCSC build danRer7 (lck:GFP^+^ RNA Seq) or GRCz10 (CD8:GFP^+^ RNA Seq) of zebrafish genome using Tophat 2.0.11^49^ without novel splicing form calls. Transcript abundance and differential expression were calculated with Cufflinks 2.2.1.^50,51^ FPKM values were used to normalize and quantify each transcripts; the resulting list of differentially expressed genes are filtered by log fold change > (0.05) and q-value > (0.05).

#### Assay for Transposase Accessible Chromatin (ATAC-seq)

50,000 lck:GFP^+^ cells were sorted from thymi of 8 months old (juvenile) adult Tg(*lck*:GFP) zebrafish at purity of above 95%. Cells were washed once with 50 μl of cold 1X PBS and spin down at 500 × g for 5 min, 4°C. After discarding supernatant, cells were lysed using 50 μl cold lysis buffer (10 mM Tris-HCl pH 7.4, 10 mM NaCl, 3 mM MgCl2, 0.1% IGEPAL CA-360) and spun down immediately at 500 × g for 10 mins, 4°C. Then the cells were precipitated and kept on ice and subsequently resuspended in 25 μl 2X TD Buffer, 2.5 μl Transposase enzyme (Illumina Nextera XT DNA library preparation kit, 15028252) and 22.5 μl Nuclease-free water in a total of 50 μl reaction for 1 hr at 37°C. DNA was then purified using Qiagen MinElute PCR purification kit in a final volume of 10 μl. Libraries were constructed according to Illumina protocol using the DNA treated with transposase, Sybr green, universal and library-specific Nextera index primers. The first round of PCR was performed under the following conditions: 72°C, 5 min; 98°C, 30 sec; [98°C, 10 sec; 63°C, 30 sec; 72°C, 1 min] × 5 cycles; hold at 4°C. Reactions were kept on ice and using a 5 μl reaction aliquot, the appropriate number of additional cycles required for further amplification was determined in a side qPCR reaction: 98°C, 30 sec; [98°C, 10 sec; 63°C, 30 sec; 72°C, 1 min] × 20 cycles; hold at 4°C. Upon determining the additional number of PCR cycles required further for each sample, library amplification was conducted using the following conditions: 98°C, 30 sec; [98°C,10 sec; 63°C, 30 sec; 72°C, 1 min] × appropriate number of cycles; hold at 4°C. Libraries prepared went through quality control analysis using an Agilent Bioanalyzer. Samples with appropriate nucleosomal laddering profiles were selected for next generation sequencing using Illumina Hiseq 2500 platform.

#### ATAC-seq analysis

All zebrafish ATAC-Seq datasets were aligned to build version Zv9/danRer7 of the zebrafish genome using Bowtie2 (version 2.2.1)^53^ with the following parameters: –end-to-end, -N0, -L20. We used the MACS2 version 2.1.0^54^ peak finding algorithm to identify regions of ATAC-Seq peaks, with the following parameter –nomodel –shift −100 –extsize 200. A q-value threshold of enrichment of 0.05 was used for all datasets.

#### Imaging of still images

Gross fluorescent images were taken using a fluorescent stereo microscope Leica M165 FC equipped with a 20 megapixel color CMOS camera DMC5400, and using Leica LAS X software. Confocal Images of sorted CD8α:GFP^+^ lymphoid T cells and myeloid DCs were obtained on an LSM 880 microscope (ZEISS, Thornwood, NY). GFP was excited sequentially with a 488 nm laser line. Fluorescence was captured with a 63× 1.4 NA oil immersion objective. Laser excitation light passing through the sample was captured by a 0.55 NA condenser equipped with DIC optics and imaged onto a PMT detector to produce an overlaid DIC image. To image zebrafish melanoma for still live imaging, the zebrafish were euthanized in 4g/L Tricaine, fixated on 35×10 mm tissue culture dish (Falcon), immersed with PBS and put directly under the scope without further manipulation. Confocal in vivo imaging of melanoma was performed on an Axio Examiner upright microscope (ZEISS, Thornwood, NY) equipped with an Insight DS+ tunable laser (Spectra-Physics). EGFP and mCherry were excited simultaneously with 930 nm light. Fluorescence was captured with a 10× 0.5 NA W Plan-Apochromat water dipping objective and imaged onto non-descanned GaAsP detectors. Image stacks were taken with a 2 mm spacing.

Second harmonic generation (SHG) Imaging to detect collagen was performed on an upright AxioExaminer microscope with LSM 980 scanhead and a two-channel non-descanned BiG.2 detector (Carl Zeiss Microscopy, Whiteplains, NY). SHG signal was generated with an InSight X3+ IR laser tuned to 930 nm. The back-scattered signal was collected by a 10× 0.5 NA W Plan-Apochromat objective and imaged through a 460–500 nm bandpass filter.

Images were processed using the Imaris software (Oxford Instruments), followed by minor brightness and contrast corrections applied to the entire image, to allow clear visualization, if needed, using photoshop software (Adobe).

#### Time-lapse live imaging

To maintain the zebrafish alive for 15 hours, a water flow system was built based on Xu et al.,^9^ with modifications as follows. 0.75x Tricaine (1g/8L system salted water+E3+Tris pH 9.0) was pumped using Ismatec REGLO Digital 4-channel 12 Roller variable speed pump (Cole Parmer, USA) in a constant flow rate of 6 ml/minute into the mouth of intubated zebrafish placed on custom-made stainless-steel plate with an orifice to allow suction of excess of water during the imaging period. The zebrafish was anesthetized and stabled using 1% agarose dissolved in salted water before intubation. Throughout the imaging time, excess of water was suctioned out using a KNF N86 KTP vacuum pump (Cole parmer, USA). Water temperature was kept constant at 28°C using SH-27B in-line heater and controlled by TC-324 heater controller (Harvard Apparatus, USA). The pressure at the output was measured using ProSense digital pressure sensor (AutomationDirect, USA), and a feedback loop to the peristaltic pump using Arduino Uno microprocessor was used as a safety mechanism in case of clogging. Time-lapse live imaging was obtained using ZEISS LSM 980 scanhead coupled to an Axio Examiner upright microscope (both, ZEISS,Thornwood, NY) and equipped with an Insight DS+ tunable laser (Spectra-Physics) as described above. 3D Images were acquired every 6–8 minutes for 20 hours with stacks taken with a 2 μm spacing.

#### Analysis and quantification of 3D images

Image processing was performed using Arivis Vision4D 3.4–3.6. For the segmentation of live 3D images two dedicated pipelines were used, one for the segmentation of the tumor and CRATERs, and another for T-cells. When treatment vs. control, or gene KO vs, wild type samples were quantified, the analysis was done blind to the condition of the analyzed sample, by a different person (AT) than the one collecting the data (AL or GLS). For the tumor and CRATERs, bright mCherry cells were enhanced using the “Normalization” and segmented using “Blob finder” tools in Arivis Version4D in order to exclude them from further analysis. For tumor segmentation Gaussian noise reduction was used followed by “Otsu” thresholding method. For the segmentation of CRATERs a black top-hat transform was performed on the tumor segment, in short, morphological closing with a 40 voxels square kernel, followed by subtraction of the tumor segment from the morphological closing result, an additional step of morphological opening with a 2 voxels kernel was performed to clean up residual segments arising from the mismatch between the tumor morphology and the square kernel. For T-cells segmentation Gaussian noise reduction followed by “Blob finder” was performed on the GFP channel. All resulting segmentation were visually inspected and manually corrected if necessary. Resulting dimensions, distance calculations and an image of the segmented tumor and CRATERs were exported to Python for calculations of tumor coverage and T-cells density, calculated for CD8^+^ cells in direct contact with segmented CRATER area or tumor area.

#### FACS sorting of T cells for scRNA-seq

Melanoma tumors from 3 Tg(cd8α:EGFP;*lck*:mCh) fish injected with tumor constructs using the MAZERATI approach^24^ (described above) and normal skin from 2 Tg(*cd8α*:EGFP;*lck*:mCh) fish were dissected and processed as previously described. Lysis buffer was prepared containing per reaction: 125U RNase inhibitor (Takara Bio, 2313A), 2.5mM dNTP (NEB, N0447S), 2.5 μM oligo-dT primer (IDT, 5^’^AAGCAGTGGTATCAACGCAGAGTACT30VN-3^’^), 0.05% Triton X-100 (Sigma-Aldrich, 93443), and nuclease-free water (Ambion, 9937) to 4 μl. 96-well PCR plates (Bio-Rad, HSP9601) were loaded with 4 μl lysis buffer per well, sealed with Aluminum Foil-96 One Tab seals (VWR, 60941–126), and placed on ice. Cells were stained with Sytox Red (Thermo Scientific, S34859) immediately before FACS-sorting individual live cells in the 96-well plates on a FACs Aria III (Becton Dickinson), as described above. 6 plates of cd8α:EGFP^+^l/ck:mCh^+^ cells were sorted from tumors (3 plates from fish 111–1, 2 plates from fish 125–2, and 1 plate from fish 107–24), for a total of 576 tumor-derived cells. 2 plates of cd8α:EGFP^-^/lck:mCh^+^ cells were sorted from normal skin, for a total of 192 cells from normal skin (1 plate from fish N1, 1 plate from fish N2). Plates were spun down and stored at −80°C immediately after sorting.

#### SMART-seq library generation

cDNA synthesis was performed using the SMART-seq2 protocol.^69,70^ In brief, 96-well plates containing single-cell lysates were thawed on ice followed by first-strand synthesis. 6 μl of reaction mix (16.7 U μl^−1^ SMARTScribe Reverse Transcriptase (Takara Bio, 639538), 1.67 U μl^−1^ Recombinant RNase Inhibitor (Takara Bio, 2313B), 1.67X First-Strand Buffer (Takara Bio, 639538), 1.67 μM TSO (Exiqon, 5′-AAGCAGTGGTATCAACGCAGAGTGAATrGrGrG-3′), 8.33 mM dithiothreitol (Bioworld, 40420001–1), 1.67 M Betaine (Sigma, B0300–5VL) and 10 mM MgCl_2_ (Sigma, M1028–10X1ML)) was added to each well. Reverse transcription was carried out by incubating wells on a thermal-cycler (Biorad) at 42°C for 90 min, and stopped by heating at 70°C for 5 min. Subsequently, 15 μl of PCR mix (1.67X KAPA HiFi HotStart ReadyMix (Kapa Biosystems, KK2602), 0.17 μM IS PCR primer (IDT, 5′-AAGCAGTGGTATCAACGCAGAGT-3′), and 0.038 U μl^−1^ Lambda Exonuclease (NEB, M0262L) was added to each well, and second-strand synthesis was performed using the following program: 1) 37°C for 30 min, 2) 95°C for 3 min, 3) n cycles of 98°C for 20 s, 67°C for 15 s and 72°C for 4 min, and 4) 72°C for 5 min, with n=23 for the plate from fish N1 and n=26 for the plates from fish 111–11 and 107–24. The plates from fish 125–2 and N2 required >26 cycles of preamplification, suggesting low-quality RNA input and thus were excluded from further analysis. An AMPure XP bead (Beckman Coulter, A63882) clean-up was performed according to manufacturer instructions, with bead:sample ratio.7X. The amplified product was diluted with a ratio of 1 part cDNA to 20 parts EB (Qiagen, 1014609), and concentrations were measured with a dye-fluorescence assay (Quant-iT dsDNA High Sensitivity kit; Thermo Fisher, Q33120) on a SpectraMax i3x microplate reader (Molecular Devices). cDNA was diluted in EB to final per-well concentration of approximately 0.25 ng/μl. Illumina sequencing libraries were prepared using 0.4 μl of cDNA from each sample well, as described previously.^71^ Libraries were sequenced on the NovaSeq 6000 Sequencing System (Illumina) using 2 × 100-bp paired-end reads and 2 × 12-bp index reads with either a 200- or 300-cycle kit (Illumina, 20012861 or 20012860).

#### scRNA-seq data processing

Quality control of SMART-Seq datasets was performed by FastQC (http://www.bioinformatics.babraham.ac.uk/projects/fastqc/) and Cutadapt^52^ to remove adaptor sequences and low-quality regions. The high-quality reads were aligned to a Zon lab custom genome^8^ using STAR 2.7.0 Spliced Transcripts Alignment tool.^55^

Analysis was performed using Seurat v4.0.2 and R v4.0.4. Cells with fewer than 500 or greater than 3000 features and cells with greater than 10% mitochondrial reads were excluded from analysis. Cells were split into Normal and Tumor samples and batch corrected using Seurat scRNA-seq integration^58^ with 2000 integration features. Principal component analysis, clustering, and UMAP dimensionality reduction was performed using Seurat with default parameters and selection of 15 dimensions, based on plotting the percentage of variance explained by each principal component, and a resolution of 0.8. Differentially expressed gene markers were identified using the Seurat function FindAllMarkers with a minimum percent expressing cells of 0.25 and the default log-fold change threshold of 0.25. Genes included in expression plots not identified as cluster-specific markers: bcl11ba, TCRa.C, TCRb. C1, trdc, tcrg, mmp9, lyz, cd8β, and cd8α.

#### Tissue preparation and FACS for vascular cells scRNA-seq

We selected an adult Tg(fli1ep:dsRedex, flk1:EGFP) zebrafish bearing an mitfa:BFP^+^ tumor generated by the MAZERATI system (injection with 1) a MiniCoopR vector expressing an mitfa mini-gene and BRAFV600E, 2) a CRISPR MiniCoopR vector expressing an mitfa mini-gene, mitfa-driven Cas9, a gRNA targeting Tyrosinase (GGACTGGAGGACTTCTGGGG), and 3) a gRNA targeting p53 (GGTGGGAGAGTGGATGGCTG), and a vector expressing BFP under the mitfa promoter). The fish bore a protruding tumor with tumor-adjacent skin that presented as normal skin (lacking BFP^+^ cells or dark pigmented melanocytes). The fish was euthanized by rapid cooling in ice water. The tumor and healthy skin were independently harvested and transferred to HBSS medium (Gibco). The tissue was dissociated using a size 11 surgical blade, then incubated for 30 minutes in 0.15mg/mL Liberase (Sigma cat. no. 05401119001) in HBSS while shaking at 150–200rpm at 37C. The solution was resuspended by pipetting halfway through the 30 minute incubation. FBS was added to 10% and cells were passed through a 40um filter (additional 500uL HBSS+2%FBS was passed through to rinse filter) and pelleted by centrifugation at 470g for 5 minutes before resuspension in HBSS+2% FBS. Cells were stained with Sytox Red (Thermo Scientific, S34859) as a live/dead stain (Abcam) and sorted on a FACs Aria III (Becton Dickinson) using the FACSDiva software (Becton Dickinson). Gates were drawn using negative and single-fluorescent reporter control zebrafish. FACS index data was recorded during sorting. Fli1a:dsRed^+^;Flk1:GFP^+^ and Fli1a:dsRed^+^;Flk1:GFP- cells were sorted from both tumor and normal skin directly into 384-well capture plates (Single Cell Discoveries) and immediately spun down 1000xg for 1 min at 4C before being put directly on dry ice and promptly stored at −80°C.

#### Sequencing and Analysis for vascular cells

Single-cell RNA sequencing was performed by Single Cell Discoveries and sequenced by CEL-Seq2 as previously described.^72^ The data was mapped by Single Cell Discoveries to Zv11 with the addition of 5 sequences: BRAFv600e, TagBFP, dsRed, mCherry, and EGFP. Analysis was performed using Seurat v4.1.0 and R v4.0.4. Cells with fewer than 50 or greater than 2000 features, fewer than 100 RNA count, greater than 10% mitochondrial reads, or greater than 70% ERCC spike-in reads were excluded from analysis. Cells were split by the plate in which they were sorted and batch corrected using Seurat scRNA-seq integration SCTransform^58^ using the default 3000 variable features. Principal component analysis, clustering, and UMAP dimensionality reduction was performed using Seurat with default parameters and selection of 30 dimensions, based on plotting the percentage of variance explained by each principal component, and a resolution of 1.0. Differentially expressed gene markers were identified using the Seurat function FindAllMarkers with a minimum percent expressing cells of 0.25 and the default log-fold change threshold of 0.25. Genes included in DotPlot expression plot are statistically significantly enriched in the specified cluster[s] (adjusted p-value <1e-9) excepting: krt91 (cluster 0 had no statistically significant marker genes and appeared to represent a mixed population), sox7, and fli1a.

#### In situ transcriptome processing via Slide-seqV2

For Slide-seq assay, whole zebrafish bearing melanoma tumors were frozen in OCT on dry ice and placed at −80°C. Slide-seq arrays were prepared and spatial bead barcodes sequenced following Slide-seqV2^22^ protocol using arrays created with custom synthesized barcoded beads (5’-TTT_PC_GCCGGTAATACGACTCACTATAGGGCTACACGACGCTCTTCCGATCTJJJJJJJJTCTTCAGCGTTCCCGAGAJJJJJJNNNNNNNVVT30–3’) with a photocleavable linker (PC), a bead barcode sequence (J, 14 bp), a UMI sequence (NNNNNNNVV, 9 bp), and a poly dT tail.

OCT-embedded frozen tissue samples were warmed to −20°C in a cryostat (Leica, CM3050S) and serially sectioned at a 10 μm thickness (2–3 Slide-seq array replicates per sample), with consecutive sections used for hematoxylin and eosin staining and immunofluorescence staining. Each tissue section was affixed to an array and moved into a 1.5 mL Eppendorf tube for downstream processing. The sample library was prepared as previously described.^22^

Libraries were sequenced using the following read structure on a NovaSeq (S2; Illumina): Read1: 42 bp; Read2: 41 bp; Index1: 8 bp, and sequences were processed as previously described^22^ using the pipeline available at https://github.com/MacoskoLab/slideseq-tools.

For CRATER vs non-CRATER analysis, RCTD was applied as described^73^ to identify cell types. We marked CRATER and non-CRATER areas (shown in Figure 2A, circles) on the expression map and applied C-SIDE to perform differential expression analysis as described.^74^

#### Quantification of Zebrafish Tumor Size

Images of tumor-bearing zebrafish were captured before treatment and 7 days after treatment using a Samsung Galaxy A54 smartphone. To determine changes in tumor size, tumors were manually segmented using QuPath software.^48^ The 2D area of each segmented tumor was then extracted using QuPath built-in measurement functionalities. Pixel size was normalized across different images using common features in the images.

#### B2M CRISPR mutagenesis

Target selection for CRISPR/Cas9-mediated mutagenesis was performed using CHOPCHOP.^75^ The selected sgRNA for *b2m*, TAAATCCAAACCGGGCAGCG having GC-content 55%, self-complementarity=0,MM0= 0, MM1=0, MM2=0, MM3=0 and predicted efficiency of ∼ 69% of editing. The sgRNA templates were generated using the protocol as previously described.^76^ mMESSAGE mMACHINETM SP6 Transcription Kit (Invitrogen, AM1340). The gRNAs were validated using T7 endonuclease I assay in 72 hpf embryos. Primers used for detection of deletion of *b2m* are: Forward: 5’-CATCTTCGCAATACCTTAGGCT-3’ and reverse: 5’-TCAGCTAAGGTAAGTGAACAGGCCTTAATTTGGAC-3’. The selected guides were integrated into a MAZERATI vector with Cas9 expressed under the mitfa promoter allowing tissue specific B2M CRISPR in melanoma.^24^ Tumor grown in B2M CRISPR vector or Tyrosinase CRISPR vector as control were imaged using upright confocal. Following imaging, the tumors were resected and evaluated for editing efficiency by deep sequencing of PCR amplicons. In brief, genomic DNA from zebrafish tissue was extracted with DNeasy blood and tissue kit (Qiagen, 69506). The CRISPR loci of the targeted genes were amplified using the primers above and barcoded with the Illumina NGS adaptor. Amplification was performed using Phusion^®^ High-Fidelity PCR Master Mix with HF Buffer (NEB, M0530), with the following conditions: 98°C 3 minutes, [98°C 10 seconds, T annealing 10 seconds, 72°C 10 seconds] × 35 cycles, 72°C 5 minutes. T annealing was 63°C]. Then, PCR amplicons were purified on PCR purification kit columns prior to sequencing. For the analysis the sequencing reads were first trimmed for quality and aligned to the GRCz11/danRer11 assembly using Bowtie2^16^ with the –very-sensitive setting. Mutations were quantified by the R software CrispRVariants-version 1.20^59^ using a minimum read count of 20. The R version in this analysis was 4.1.0 (2021–05-18).

#### CpG ODN treatment

CpG ODN 2007 (Invivogen, USA) was dissolved in sterile water to stock concentration of 2μg/μl. CpG ODN was further diluted using PBS to 1μg/μl. For control, sterile water were diluted in PBS at the same ratio as CpG ODN. Either control or CpG ODN solution was injected directly into the tumor at a volume of 1μl using 10 μl Hamilton syringe model 1701 (33s/0.6’/2). CpG ODN or control were injected daily and tumors were analyzed 24 hours post injection.

#### TGF-β inhibition treatment

SB431542 (Sigma Alderich, USA) was used to inhibit TGF-β. SB431542 was dissolved in DMSO to 10mM. SB431542 was further dissolved in fish water to a concentration of 10μM. To achieve TGF-β inhibition, zebrafish containing tumors were immersed in fish water contained 10 μM SB431542 and analyzed 24 hours after treatment. As control, zebrafish were immersed in fish water containing DMSO 0.1%.

#### RNAscope

Zebrafish tumors were excised and were immediately frozen in OCT in 2-methylbutane over liquid nitrogen or ethanol 100% over dry ice. Samples of 10μm were cut using cryostat (Leica) and placed on superfrost histological slides (Fisher scientific, USA) and stored in −80°C until use. RNAscope was performed using RNAscope^™^ Fluorescent multiplex assay (ACDBio, USA) according to manufacturer’s instructions for fresh frozen samples. RNA probes for mCherry (RNAscope-Probe-mCherry, C2 channel, stained with Atto647N), zebrafish CD8α (RNAscope^®^ Probe- Dr-cd8a-C3 channel, stained with Atto550) were used to detect the melanoma and CD8^+^ cells. In addition, costume probes designed for the zebrafish genes ifng, b2m and zbtb46 were used at the C1 channel, stained with AF488. Slides were imaged using an inverted AxioObserver with LSM 900 scanhead microscope, and equipped with 405, 488, 561 and 640 nm diode lasers. Images were prepared using Imaris software (Oxford Instruments).

#### Analysis and Quantification of RNAscope Images

RNAscope 3D image stacks were first converted to 2D images using a maximum intensity Z-projection. All subsequent quantification and analyses were performed on these projected images.

Due to high nuclear density within the tumor, which prevented accurate nuclear segmentation, CD8 expression was used for the identification and segmentation of CD8^+^ cells. The CD8 channel image data were background corrected. Initial CD8^+^ foci were segmented using the “Blob finder” tool in Arivis Vision4D, and their spatial coordinates were exported for analysis in Python. To filter out non-specific signals and regions of low CD8 expression, Density-Based Spatial Clustering of Applications with Noise (DBSCAN) was applied. This clustering preserved only foci possessing at least two other foci within a 10 μm radius. The filtered foci coordinates were then used to generate a grayscale density map using Gaussian Kernel Density Estimation (KDE). This KDE-generated density map was then imported back into Arivis Vision4D as a new image channel, which served as the basis for the segmentation of CD8^+^ cells using the “Blob finder” tool. For the analysis of interferon expression, the ifng channel was background corrected, and ifng foci were segmented using the “Blob finder” tool.

Tumor regions were segmented based on the 562 nm channel, which captured both mCherry protein signal and mCherry-specific RNAscope probe signals. This segmentation was performed in Arivis Vision4D using a membrane-based segmentation algorithm. A morphological closing operation was applied to fill small gaps within the segmented tumor mass. The tumor edge was defined as a 15 μm-wide border, created by dilating the primary tumor segmentation by 15 μm and then subtracting the original tumor segment from this dilated area.

For each identified cd8a cell, its shortest distance to the tumor and to the tumor border, and the total number of associated ifng foci were quantified. These quantitative data were exported to Python for further statistical analysis and data visualization, utilizing the scikit-learn and seaborn libraries.

#### Whole mount TUNEL assay

TUNEL assay was performed using Click-iT^™^ Plus TUNEL Assay Kits for In Situ Apoptosis Detection (Fisher Scientific, USA) on whole tumors. Tumors were excised with wide margins from the zebrafish and incubated in 4% PFA in PBS (Boston BioProducts, USA) for 15 minutes. Samples were washed with BSA 3% in PBS twice and incubated in proteinase K solution for 20 minutes at room temperature. Washed the samples with PBS and immersed in TdT reaction buffer for 10 minutes in 37°C, then removed the reaction buffer and added the TdT reaction mix, prepared according to protocol for 500 μl to cover the samples. Incubated for 1.5 hours in room temperature. Washed the slides with 3% BSA and 0.1% Triton-X-100 in PBS for 5 minutes, then washed with PBS. To stain, added Click-iT Plus TUNEL reaction cocktail prepared according to manufacturer’s instructions for 500 μl solution and incubated for 60 minutes in 37°C incubator. Washed with 3% BSA in PBS for 5 minutes and stored at 4°C. Samples were imaged the next day in PBS using an Axio Examiner upright microscope (ZEISS, Thornwood, NY) equipped with an Insight DS+ tunable laser (Spectra-Physics). Fluorescence was captured with a 10× 0.5 NA water dipping objective and imaged onto non-descanned GaAsP detectors. To assure the signal report TUNEL^+^ cells, tumors of zebrafish underwent the same staining procedure, except that PBS was added to the TdT reaction mixture instead of the TdT enzyme. Only low background signal was detected in this tumor.

#### Murine melanoma transplantation and adoptive CD8^+^ T cells transfer

The UV2 mouse melanoma cell line^77^ was transduced with lentivirus carrying ovalbumin (OVA) gene, and Zsgreen^+^ cells were sorted by flow cytometry. 400,000 cells were resuspended in 100 ul PBS and injected subcutaneously into 8 weeks Rag1^(−/−)^ mice. CD8^+^ T cells were using the Mouse CD8^+^ T Cell Isolation Kit (StemCell Technologies) and activated with Dynabeads^™^ Mouse T-Activator CD3/CD28 for T-Cell Expansion and Activation (ThermoFisher scientific) according to the manufacturer’s protocol. Seven days post melanoma transplantation, 1 million activated CD8^+^ T cells, either OVA sensitive (OT) or wild type, with no specific OVA sensitivity, were transferred by tail vein injection into the tail vein of the tumor bearing mice. Five days post CD8^+^ T cells adoptive transfer, the mice were sacrificed using CO2 asphyxiation and the tumors were harvested. One lobe of each tumor was separated for whole mount 3D analysis. The rest of the tumors were put in fresh 4% PFA for two days, changed to ethanol 70% until floated, then frozen over ethanol 100% over dry ice, embedded in OCT.

#### Murine tumor immunofluorescence staining

7u sections were cut from the PFA fixated frozen samples of murine tumors using Leica cryostat.

The sections were washed with PBS, then stained with Rabbit anti-CD8α (abcam) and APC anti-mouse β2-microglobulin Antibody (Abcam), both 1:200, at room temperature for one hour, followed by x3 PBS wash and incubation with Rhodamine (TRITC) AffiniPure^™^ Goat Anti-Rabbit antibody for CD8α detection, for 1 hour. The samples were then washed x3 with PBS, stained using Hoechst 33342 (Biotium) and closed using Fluoromount Aqueous Mounting media (Sigma).

#### Analysis of Mouse Tumor Images

Images from tumor of untreated and wild type CD8^+^ transferred mice were segmented using the same methodology described above for zebrafish image analysis. In contrast, images from OT transferred presented tumors that were too sparse to permit direct segmentation as a contiguous mass. Therefore, an alternative strategy was used to quantify tumor attrition in these samples by estimating the pre-treatment tumor boundary from the distribution of remaining tumor fragments. For OT samples, residual tumor components within the 3D image stacks were first segmented as discrete ’blobs’ using the “Blob finder” tool in Arivis Vision4D. The resulting 3D labeled image was exported to Python for further analysis. In Python, the centroid coordinates of each segmented tumor blob were extracted. A polynomial surface was then fitted to these coordinates, the optimal degree for this polynomial surface (ranging from 2 to 10) was determined empirically for each sample by selecting the degree that resulted in the best fit to the spatial distribution of the blobs; this was consistently found to be either a 5th or 6th order polynomial.

This fitted polynomial surface served as an estimation of the original tumor surface area. The segmented tumor blobs were then projected onto this estimated surface. Tumor attrition was quantified as the percentage of the surface area that was not occupied by the projection. Custom Python scripts developed for this surface fitting and attrition analysis are available from the upon request.

#### Quantitative analysis of CyCIF processed human melanoma samples

CyCIF data was procured from database collected at Nirmal et al.,^34^ made available for us by AJN and PKS. The data was analyzed to identify CRATERs in human melanoma and immune cell characteristics related to CRATERs. Preprocessing and segmentation of the CYCIF images were performed as follows. Prior to segmentation, the membrane labeled channels (CD4, CD8a, CD163, CD3d, CD11c, MCAM, ICOS, HLA-A, CD31, MART-1, CD16) and the cytoplasmic label S100A were each normalized to their 99^th^ percentile value and summed. The Nucleus channel was normalized separately. Segmentation was then performed using Cellpose^61^ with the TissueNet model, allowing identification and segmentation of individual cells (Figure S5E). For gene expression data, background subtraction was performed on each channel using a rolling ball algorithm with a radius of 50 pixels. Mean fluorescence intensity per cell was then extracted from each channel for further analysis. Clustering and analysis of the single cell data was performed using the Scanpy^62^ python package. For cell type identification, the following marker genes were utilized: SOX10, CD163, CD3d, CD8a, FOXP3, CD11c, CD4, CD68, CD31, Granz.B, and MART1. The log transformed gene expression data was scaled using standard scaling prior to dimensionality reduction. Embedding of the data was performed using UMAP^63^ and clustering was done with Leiden clustering.^64^ The clustered data was then manually inspected and corrected. Clusters were then classified to specific cell types (Figure S5F).

CRATER regions were manually labeled based on the S100A, CD8a, CD31 and αSMA channels. Segmentation of the tumor mass was performed by applying Otsu thresholding to the S100A channel, followed by morphological closing to fill small holes within the tumor region, and morphological opening to remove small, disconnected tumor fragments. The tumor margin region, enclosing the S100a^+^ cell mass, was segmented by dilating the tumor segmentation and subtracting the original tumor area, defining the boundary area (Figure S5D).

To calculate cell densities, cells overlapping the border region but not CRATERs were classified as border cells. Tumor cells were defined as overlapping the tumor segmentation without overlapping CRATERs or the border. This enabled quantitative analysis of cell densities across different spatial regions.

#### Human sample acquisition and multiplexed immunofluorescence

Tumor samples were collected from cancer patients receiving clinical immunotherapies at Dana-Farber Cancer Institute. All individuals gave written informed consent to utilize their leftover tumor samples in accordance with the Institutional Review Board (IRB) approved protocol 17–000. Staining was performed overnight on a BOND RX fully automated stainer (Leica Biosystems). Tissue sections of 5-μm thick FFPE were baked for 3 hours at 60°C before loading into the BOND RX. Slides were deparaffinized (BOND DeWax Solution, Leica Biosystems, Cat. AR9590) and rehydrated with series of graded ethanol to deionized water. Antigen retrieval was performed in BOND Epitope Retrieval Solution 1 (pH 6) (Leica Biosystems, Cat. AR9961, AR9640) at 95°C. Deparaffinization, rehydration and antigen retrieval were all pre-programmed and executed by the BOND RX. Next, slides were serially stained with primary antibodies. Each primary antibody was incubated for 30 minutes. Subsequently, anti-mouse plus anti-rabbit Opal Polymer Horseradish Peroxidase (Opal Polymer HRP Ms + Rb, Akoya Biosciences, Cat. ARH1001EA) was applied as a secondary label for a 10 minute incubation. The corresponding Opal Fluorophore Reagent (Akoya) was applied for 10 minutes. Slides were incubated in Spectral DAPI solution (Akoya) for 10 minutes, air dried, and mounted with Prolong Diamond Anti-fade mounting medium (Life Technologies, Cat. P36965) then stored in a light-proof box at 4°C prior to imaging. Image acquisition was performed using the Vectra Polaris multispectral imaging platform (Vectra Polaris, Akoya Biosciences). The target antigens, antibody clones, dilutions, and antigen retrieval conditions are listed in the above table. Images were either assessed pathologically or underwent quantitative analysis.

#### CRATER identification in human samples

CRATERs in human melanoma follow the below criteria:

Location: at the borders of stromal and melanocytic compartments. Either at the peripheral tumor/stromal interface/border or at the perivascular stromal/melanoma interface.

size: 20–50um.

Distinct features to distinguish from perivascular area (PVA) invagination: A CRATER is a gap of staining in a tumor that is surrounded by melanoma cells and forms a discrete excavation along an otherwise relatively regular tumor/stromal interface. CRATERs contain one or two collagen fibers, but not more, as is the case for the intervening tumor/stromal interface. There is low nuclear density in the CRATER compared to the perivascular stroma. There are no blood vessels within the confines of the gap, and no CD105^+^ stromal or endothelial cells. There are no pericytes that stain with aSMA.

**Additional markers include** elevated expression of MART-1, PD-L1 and HLA-A. These markers highlight the CRATERs well in immunostaining (Figure 6).

The composition of cells can aid to finally confirm the structure as CRATERs. CRATERs harbor one or two CD163^+^ dendritic cells and do not harbor CD4^+^ T cells (including Treg).

All the above will enable a clear identification of a CRATER.

#### Evaluation of variability in CRATERs segmentation between different annotators

To assess the variability and reproducibility of CRATERs segmentation, three independent annotators segmented an identical, predefined sample according to the above CRATER identification guidelines. The resulting segmentation masks were then compared pairwise to quantify the level of agreement using the F_1_ score.

For any two segmentation masks, a segmented CRATER in the first mask was considered a match to a CRATER in the second mask if their overlap exceeded a defined threshold. The overlap was quantified using the Jaccard Index (JI), defined as the Intersection over Union (IoU):

$$JI(A,B)=\frac{A\;\cap B}{A\;\cup B}$$

A JI score greater than 0.4 was set as the threshold for a successful match, the choice of threshold was done by examining the JI histogram which showed that all JI lower than 0.4 were 0. Based on this criterion, True Positives (TP) were defined as matched CRATERs between the two masks. False Positives (FP) were CRATERs in the first mask with no match in the second, and False Negatives (FN) were CRATERs in the second mask with no match in the first.

These values were used to calculate Precision and Recall, and subsequently the F_1_ score, which is their harmonic mean:

$$F_{1}=2*\frac{\;Precision\;\;*\;Recall\;}{\;Precision\;+\;Recall\;}$$

$$Precision\;=\frac{TP}{TP+FP}$$

$$Recall\;=\frac{TP}{TP+FN}$$

The F_1_ scores from all three pairwise comparisons were then averaged. The resulting mean F_1_ score was 0.76, indicating a high degree of agreement between annotators.

#### Quantitative analysis of multiplexed immunofluorescence processed human melanoma samples post treatment

Prior to all image analysis and CRATER scoring, each image was given a random unique identifier and then scored blinded to sample ID and patient outcome. Identification of perivascular border was performed in a semi-automated manner using Arivis4D and QuPath. In short, The S100a channel was filtered using a median filter, followed by thresholding and segmentation of the tumor mass. The segmented area was subject to morphological closing, followed by subtraction of the non-closed segment from the closed one, resulting in segmentation holes in the tumor mass. These holes were then filtered based on size and CD31 expression to identify perivascular area. Segmentation maps were then transferred to QuPath for manual correction and manual CRATER identification. CRATERs were annotated manually on QuPath, according to CRATER definition guidelines delineated below, excluding the additional markers of PD-L1, MART-1,HLA-A and immune cells. Per each sample, all perivascular-melanocyte boundaries and stromal borders in the sample were examined for CRATER presence. Upon detection of CRATER, the area was annotated manually and was designated a “CRATER” annotation class. CRATER annotation was done primarily by one person and then re-evaluated in a blinded manner by three more people, with F_1_ concordance value of 0.76, calculated as described above. Perivascular boundary length was calculated using QuPath and CRATER density within the tumor was calculated as CRATER number per linear 1cm length of tumor margin. Areas of massive fibrosis were omitted from the analysis due to the inability to determine a clear stromal-tumor border. Intratumoral CD8^+^ T cells density was measured and calculated at the pathology department of Brigham and Women’s Hospital (BWH), as part of sample processing for pathological evaluation. The values reflect the average of CD8^+^ T cells number per mm^2^ in 6 fields of view per sample and their corresponding standard error. CRATER density was correlated to the clinical outcome determined as closely as possible to the biopsy collection. The clinical outcome was evaluated clinically by radiological assessment, based on presentation of durable clinical benefit (e.g. a sustained BOR of SD, PR, or CR per RECIST v.1.1) or lack of, for at least two restaging scans. If progressive disease was noted after a given treatment, meaning that the tumor kept growing despite the patient undergoing cycles of immunotherapy, the outcome was considered “non responsive” in this study. Otherwise, the outcome was considered responsive.

#### In vivo Reflectance confocal microscopy (RCM) imaging in human patients

In vivo patient imaging was conducted on melanoma lesions in 2 patients for clinical diagnosis or treatment monitoring after receiving T-VEC injection. Written informed consent was obtained where necessary. Imaging was performed using an RCM device (tissue-coupled VivaScope 1500 or handheld VivaScope 3000, Caliber I.D., Rochester, NY). RCM images were acquired at multiple locations within the lesion for comprehensive lesion evaluation. Between 3–4 mosaics were acquired at epidermis, dermal–epidermal junction and dermis, followed by stacks and videos. Images were read and interpreted in real-time at the bedside to select the targeted biopsy site(s) by 2 investigators (M.C. and A.S.) with over 6 years of RCM reading experience. Images were evaluated for previously described criteria for nodular melanoma.^78^ CRATERs were defined as hypo-reflective structures within or surrounding cerebriform nests (that represent melanoma tumor nests) with absence of any blood flow. Small, low to medium reflective mononuclear cells inside and at tumor periphery were termed as lymphocyte-like cells based on previous sudies.^37^

### Statistical analysis

Statistical tests were performed using Python SciPy package v1.9.3. All fields of view from the same fish were pooled together and each fish is considered as an independent repeat. We used two sided unpaired t-test for comparison between groups with normal distribution or small sample size, two sided paired t-test for comparison of the density of CD8^+^ cells in CRATERs and tumor within the same fish, and two sided Mann-Whitney U-test for groups with non-normal distribution. P < 0.05 was considered significant; *P < 0.05, **P < 0.01, ***P < 0.001.

## Supplementary Material

1 Figure S1. cd8α:EGFP transgenic zebrafish line generation and T cell-tumor infiltration, related to Figure 1 (A) Upper: cd8a gene area in ATAC-seq and bulk RNA-seq analyses of sorted lck^+^ lymphocytes from zebrafish thymi. Rectangle: area used as *cd8a* promoter.
Lower: fluorescent images of zebrafish at 1 and 6 weeks post fertilization. Arrow: EGFP^+^ zebrafish thymi. *EGFP expression in the heart is irrelevant for *cd8a* expression. It results from the backbone plasmid used to select successful transgenes.
(B) Flow cytometry analysis of Tg(cd8α:EGFP; lck:mCherry) zebrafish thymus-derived cells. Upper: FSCC plot of zebrafish thymus cells. Red population: EGFP^+^gated population appears at the lymphoid gate. Upper right image: EGFP^+^ lymphocytes sorted onto slides showing a bright EGFP^+^ cell of ∼10 μm in diameter.
Lower: lymphoid-gated flow cytometry plots of thymus, spleen, and kidney cells, showing CD8α:EGFP^+^/lck^+^ cells.
(C) T cell gene expression in bulk RNA-seq of EGFP^+^ thymus-derived sorted cells from 8-week-old (juvenile) Tg(cd8α:EGFP) zebrafish.
(D) Flow cytometry plots of thymi cells collected from WT and prkdc^(−/−)^ Tg(cd8a:EGFP ) adult zebrafish.
(E) Flow cytometry plots of melanoma-derived cells showing CD8α:EGFP^+^ cells in the myeloid gate. Right: cd8a:EGFP myeloid cells sorted on a slide.
(F and G) Bulk RNA-seq of EGFP^+^ myeloid cells sorted from melanoma tumors showing expression of genes associated with (F) CD8^+^ T and dendritic cells and
(G) myeloid cells.
(H) *In vivo* imaging of the thymic region of Tg(cd8α:EGFP) zebrafish injected intraperitoneally (i.p.) with ovalbumin-AlexaFluor 594. Left: 3D view of CD8α:EGFP dendritic cells. Right: rectangle enlarged, presenting a single plane (2 um slice), where OVA fragment is presented on the DC dendrites.
(I) Single-cell RNA-seq of sorted lck^+^ T cells, using Tg(lck:mCherry) zebrafish melanoma and normal skin, exhibit 3 clusters (a macrophage cluster, which was a contamination, was omitted from the dataset).
(J) Expression of select genes classifying T cell clusters and presenting the lack of expression of macrophage markers in the three populations presented in (I).
(K) Tissue origin labeling reveals one cluster of predominantly normal-skin-derived T cells and two clusters of primarily tumor-derived T cells.
(L) Differential gene expression analysis identifies a heterogeneous population of T cells comprising the normal skin, with tumor-derived clusters exhibiting CD8^+^, CD4^+^, and T regulatory cells.
(M) Left: confocal 3D projection of Tg(lck:mCherry) zebrafish skin, using background signal on the BFP channel to trace scale pattern. Multiple lck^+^ cells are found in the skin, many are clustered at the edge of the scales, as previously described. Middle: Tg(cd8α:EGFP; lck:mCherry) zebrafish skin. Arrows: CD8^+^/lck^+^ cells.
Right: area at the rectangle enlarged, showing overlap of cd8α:EGFP and lck:mCherry signals in T cells. CD8^+^ cells are a minority among the lck^+^ cell population in the skin.

2 Figure S2. CRATER characteristics and spatial distribution in zebrafish melanoma, related to Figure 1 (A) Left: zebrafish with an mCherry^+^ melanoma protruding behind its head (the fish is intubated for long-term time-lapse imaging). White dashed line marks the upper border of the fluorescent image (mirror image). Middle: the fluorescent image of the area marked with the white rectangle on the left image. Right: outcome of tumor and CRATER segmentation, as described in the STAR Methods, of the image in the middle. The areas lacking signal and corresponding to CRATERs are now segmented objects marked in green.
(B) Examples of CD8^+^ cells quantified as “in CRATER” and “in tumor.” Yellow arrows mark CD8^+^ cells. The cells were quantified when directly connecting the segmented areas per segmented surface (mm^2^). Some CD8^+^ cells were found in the epithelial layer covering the tumor, i.e., “outside” the tumor (upper image), and were not included in the analyses of this study, unless indicated otherwise.
(C) CD8^+^ cell distribution by distance from CRATERs, showing fewer CD8^+^ cells as the distance from CRATERs increases (*n* = 6 fish, calculated for 1,200 cells).
(D) CRATER area constitutes about 12% of the tumor surface in untreated tumors (*n* fish = 5, data are mean + SE).
(E) Snapshots of long-term live imaging. Left: low magnification of the imaged tumor. Yellow rectangle is enlarged in (K), gray rectangle is enlarged in Figure 1E.
The white rectangle to the right is enlarged in the right panel. CD8^+^ T cells (white arrows) linger in a CRATER for nearly 6 h before moving out. Time is hours: minutes.
(F) 3D image of a mitfa:BFP tumor in Tg(flk:GFP; fli1a:dsRed) zebrafish. Dashed yellow line outlines a scale protruding from the tumor mass, in the lowmagnification image. A CRATER area is enlarged at the low right corner, showing proximity to fli1a^+^/flk^−^ and flk^+^ blood vessels. White dashed line: CRATER.
(G) Single-cell RNA-seq analysis of sorted fli1a^+^/flk1^+^ and fli1a^+^/flk1^−^ cells from zebrafish melanoma and normal skin using the SORT-seq technology exhibits 7 clusters.
(H) Endothelial cell subset clustering of fli1a^+^/flk1^+^ and fli1a^+^/flk^−^ cells.
(I) Differential gene expression analysis identifies 3 endothelial cell clusters, suggestive of arterial (cluster 2), venous (cluster 3), and lymphatic (cluster 4) subpopulations.
(J) Marker genes exhibit differential expression across endothelial cell subpopulations.
(K) 2 μm slice of a z stack through the CRATER, marked by the yellow rectangle in (E). Using second harmonics generation, fine collagen fibers can be seen penetrating the CRATER from the collagen-rich capsule that encompasses the tumor (horizontal second harmonic generation [SHG] signal).
(L) 3D image of mitfa:mCherry melanoma in Tg(cd8α:EGFP; mpeg1:BFP) zebrafish. Two areas marked by rectangles 1 and 2 are enlarged on the right. Area (1): enlarged view of a CRATER harboring CD8α^+^ T cells and mpeg1^+^ cells engulfing melanoma cells (white arrows). Area (2): large CD8α^+^/mpeg^+^ cell above a CRATER.
(M) RNAscope staining for mCherry, cd8, and zbtb46 mRNA in untreated zebrafish tumor. Dashed white line: a projected CRATER area. The area, marked in a rectangle, is enlarged on the right and shown for the different channels. The arrows indicate the one nucleus associated with the zbtb46 and cd8a RNA staining.

3 Figure S3. CRATERs enlarge and harbor CD8^+^ T cells following treatment, related to Figure 3 (A) RNAscope assay detecting mRNA of mCherry, cd8a, and b2m RNAs in B2M intact and B2M KO zebrafish tumors. Representative of 5 FOV, 2 fish for each group (WT or B2M KO).
(B and C) (B) CD8^+^ cell number per field of view and (C) CRATER coverage (% of the tumor surface) in tumors of intact (WT) and melanocyte-specific B2Mdepleted melanomas. (For both graphs: dots represent fish. *n* = 4 control fish, 6 FOV, 5 B2M-depleted fish, 8 FOV. *t* test. *p* value for (B) = 0.34. *p* value for (C) = 0.12. Whiskers represent 1.5× the interquartile range).
(D) A diagram of CpG ODN treatment applied to zebrafish melanoma. Created using Biorender.com.
(E) Representative images of melanoma tumors injected daily with CpG ODN or PBS for 1, 4, and 7 days, each 24 h post last injection. Upper: WT tumor injected with CpG ODN; middle: WT tumor injected with PBS; lower: B2M KO tumor injected with CpG ODN.
(F) Fold change of tumor volume following seven daily injections of vehicle (PBS) or CpG ODN intratumorally into zebrafish. (Dots represent fish. *n* = PBS-6 fish, CpG ODN-12 fish. Whiskers represent 1.5× the interquartile range. *t* test. *p* value = 0.0001).
(G–J) (G) Relative affinity of CD8^+^ T cells for CRATERs (CD8^+^ T cell density in CRATERs/tumor), (H) CD8^+^ T cells numbers per field of view, (I) numbers per field of view of CD8^+^ T cells near or contacting tumor (<5 μmaway from tumor), and (J) numbers per field of view of CD8^+^ T cells away from or not contacting tumor (>5 μm away from tumor), all in PBS-, CpG ODN-, and tumor-specific B2M KO, CpG ODN-treated melanomas (G–J calculated for samples shown in Figures 3A and 3B: dots represent fish. *n* = PBS-5, CpG ODN-5, B2M KO CpG ODN-4 fish. 2–3 FOV per fish, data are mean ± SE. *t* test. *p value = 0.01).
(K) 3D image using second harmonic generation (SHG) microscopy to visualize collagen of CpG ODN-injected tumors in Tg(cd8α:EGFP) zebrafish (representative of 2 CpG ODN 2d- and 4 CpG ODN 4d-injected fish). CD8^+^ cell infiltration after 4 daily injections is accompanied by multiple collagen aggregations. Rectangle enlarged to the right, tilted for a side view and segmented, showing CD8^+^ cells in CRATERs and multiple collagen aggregations.
(L) Staining for mCherry, cd8a, and b2m mRNA in zebrafish tumors of WT (upper) and prkdc(^−^ /^−^ ) (lower) zebrafish, using RNAscope. The area in the rectangle in each image is enlarged to the right. The same area is shown with and without nuclei staining. Dashed white lines: projected CRATERs. b2m mRNA expression can be noted at the sides of the CRATERs (representative of 5 FOV, 2 fish for each).
(M) 3D image of melanoma in Tg(cd8a:EGFP) zebrafish treated with SB431542 (TGF-β inhibitor) or DMSO for 24 h (representative of 3 fish). Enlarged area (1): a CD8^+^ T cell cluster. (1a): a 3D view of the cluster. (1b): a single slice image at the plane beneath the cluster shown in 1a, revealing a large dendritic-shaped CD8^+^ cell beneath the cluster. Enlarged area (2): a CRATER with multiple CD8^+^ dendritic-shaped cells. Example 2: a different tumor grown in Tg(cd8a:EGFP; mpeg:BFP) zebrafish, treated with SB431542 for 24 h, shows a CD8^+^/mpeg^+^ cell (white arrows) cluster in a CRATER.
(N) CD8^+^ cell density in CRATERs vs. tumor in SB431542-treated vs. DMSO control. (*n* fish = 6 DMSO, 3 TGF-βi. Data are mean ± SE, *t* test. *p value = 0.043, **p value = 0.0012).
(O) Snapshots from 16 h long-term time-lapse imaging of mitfa:mCherry tumor in Tg(cd8a:EGFP; mpeg:BFP) zebrafish following 24 h TGF-βi treatment (Video S4).
The area marked by a rectangle is enlarged at the right. Over the course of 16 h, CD8a^+^ T cells interact with an mCherry melanoma (marked by white arrow) for nearly 9 h. They then pass it to the CD8a^+^ dendritic-shaped cell, which releases it at hour 15 of imaging, keeping a fragment of mCherry (yellow arrow). Time is hours:minutes.
(P) Image of the dendritic-shaped CD8a^+^ cell at 9 h 38 min, fluorescent color separated for mpeg (monocyte marker) and cd8α, showing expression of both mpeg and cd8a.

4 Figure S4. Murine-transplanted OVA-expressing melanoma exhibits regional tumor attrition upon CD8^+^ T cell transfer but widespread B2M expression and lack of CD8^+^ T cell specificity to areas of attrition, related to Figure 2 (A) Percent of tumor surface lacking tumor cells, measured through 3D projection of whole-mount staining of a lobe resected from OVA-expressing tumors in mice undergoing no CD8^+^ T cell transfer (UT), activated, non-OVA-sensitive, wild-type CD8^+^ T cells (WT), or OVA-sensitive CD8^+^ T cells (OT). (*n* mice: UT = 5, WT = 5, OT = 5 mean ± SE. *t* test *p value = 0.03, **p value = 0.0001).
(B)Representative images of 3D projections of tumor lobes described in (A). Left column: broad-field view. Right images: areas marked by rectangles are enlarged and shown with and without the melanoma marker. Asterisks and dashed lines: areas lacking tumor cells, resembling CRATERs in WT CD8^+^ T celltransferred tumors.
(C) Immunofluorescence staining of 2D sections of tumors following WT CD8^+^ T cell transfer. Upper: low magnification, presenting the widespread expression of B2M on the tumor surface, shown with and without melanoma marker. Lower: rectangle enlarged. An area lacking melanoma cells that resembles a CRATER is marked by dashed white line. B2M staining as well as CD8^+^ T cells can be found within the CRATERiform area and its surroundings.
(D) A representative image of immunofluorescence staining of 2D sections of tumors following OVA-sensitive CD8^+^ T cell transfer. The same area is shown for different marker combinations. Dashed white line: the tumor surface. Widespread tumor attrition and B2M expression is present. CD8^+^ T cells are found within the tumor mass rather than at the tumor surface.

5 Figure S5. Human CRATER characteristics and identification, related to Figure 5 (A) Violin plot depicting CRATERs diameter distribution in human melanoma (measured for *n* = 714 CRATERs, 2 samples).
(B and C) CyCIF images of (B) S100^+^ melanoma tissue contacting the stromal layer at the outer border of the tumor and (C) at the perivascular area. Left: low magnification. Arrows and dashed lines mark examples of CRATERs. Right: rectangle area, enlarged, presenting CRATERs containing CD8^+^ T cells and CD163^+^cells. Steps for CRATER identification, from left to right, all show the same CRATER.
(D) Perivascular area (PVA) is detected by CD31^+^ blood vessel (BV) surrounded by a layer of collagen fibers. This area will not contain S100^+^ melanoma cells. Its boundaries, i.e., perivascular-melanocytic boundaries (PMB), are marked by a yellow line. A 20 μm protrusion without melanoma cells and dense collagen is marked by a red line. It contains one fiber of collagen.
(E) Left: perivascular area (PVA), whose boundaries are marked by a yellow line, has dense nuclei content, whereas the area marked by the red line has very few nuclei. Thus, the red marked area is designated as a CRATER. The CRATER contains a CD8^+^ cell (middle) and CD163^+^ cell (right).
(F) Top: a table distinguishing PVA indentation and CRATER areas. Bottom: three additional examples of CRATERs.
(G) An example of segmentation applied on a CyCIF image, as done in this study. The image on the left is segmented on the right, highlighting the CRATER areas (yellow), tumor margins, i.e., PMBs (gray), and tumor mass (red). See STAR Methods for a detailed description of the analysis.
(H) Example of cell segmentation of a human CyCIF sample of primary human melanoma as analyzed in this study. Right: rectangle area, enlarged, to show the individual cells.
(I) Cell segmentation was followed by cell clustering and identification of cell populations stained in the sample: signal intensity heatmap of markers read in the CyCIF samples analysis, identifying cell populations.
(J) mIF followed by washing and staining of the same section with hematoxylin and eosin (H&E). A BV area containing two CRATERs is shown for both staining. In both, CRATERs are marked by red lines. Upper images: mIF staining. The rectangle area is enlarged to the right. Lower: the same area stained with H&E. The same CRATER area, marked by rectangle, is enlarged on the right. See STAR Methods for staining and analysis methods of this sample.
(K) Human cutaneous melanoma tumor sample stained for CD105 along with collagen, S100 to mark melanoma cells, CD8, and nuclei, showing expression of CD105 in blood vessels. Yellow lines: PMB.
(L) A perivascular area in which CD105 stains the blood vessels and stromal cells. Red lines: CRATERs. Yellow lines: PMB. Right: rectangle area enlarged, showing the lack of CD105 in the CRATER.
(M) Staining of mCherry (melanoma), cd8a, and endoglin (eng, i.e., CD105) mRNA in untreated zebrafish melanoma tumor (representative of 5 samples), using RNAscope. Upper: eng can be found in elongated structure in an area devoid of melanoma cells, which resemble blood vessel (BV), but not in CRATER. The area at the rectangle is enlarged on the right. Lower: two additional examples of zebrafish tumor CRATERs from different samples of untreated tumors.
(N) Representative image of a CRATER (white dashed line) extending from a perivascular area (PVA). Left: low magnification. Right: the CRATER is αSMA negative, containing CD8^+^ T cells (white arrows) expressing Tim3, Lag3, and PD-1 (representing 3 tumors).

6 Figure S6. CRATERs first appear at the vertical growth phase of melanoma development, related to Figure 5 CyCIF images of untreated primary melanoma tumors at different stages of development. For each panel, the area marked by the white rectangle in the left image is enlarged to the right. (A) Nevus.
(B) Horizontal growth, with CD8^+^ T cells aggregating around blood vessels.
(C) The tumor thickens, and multiple CD8^+^ T cells contact its margins. PD-L1 negative indentations are seen at the tumor margins.
(D) Vertical growth area forming a small tumor mass in melanoma at the vertical growth phase. CRATERs (marked by white arrows) appear at the melanoma border containing CD8^+^ T cells and PD-L1 expressing CD163^+^ DCs.
Images are representatives of the following reviewed samples: superficial radial phase: *n* = 15. Vertical growth: *n* = 3.

7 Figure S7. CRATERs expand and contain CD8^+^ T cells and apoptotic cells following treatment and may be found in lung cancer, related to Figure 7 (A) *In vivo* 3D confocal view of melanoma nests in patients using RCM. Upper: pockets akin to CRATERs can be found at the edge of a melanoma nest (enlarged).
Lower: melanoma nests in a patient treated with T-VEC. The nests are emptying of cells, and large pockets akin to CRATERs appear together with lymphocytelike cells (enlarged). White arrows indicate lymphocyte-like cells, as determined by pathologists reviewing the images (see STAR Methods).
(B) Intra-tumoral CD8^+^ T cell density value, measured at the pathology department of BWH, for each of the posttreatment samples analyzed. Graph depicts average intratumoral CD8^+^ T cell number per mm^2^ for at least 6 regions of interest per sample.
(C) Representative images of metastases resected post treatment. Left: durable response (SD). Yellow lines: PMB, arrows: CRATERs. Right: PD. Yellow arrows: CD8^+^ T cells entering the tumor. At the bottom of each image: rectangle area, enlarged. In durable response, CD8^+^ T cells aggregate in CRATERs (red line). In PD, most CD8^+^ T cells are found as singles or doublets, spread among melanoma cells.
(D) Additional representative images from durable response to ICB treatment. Upper: two additional areas from the SD sample: the same sample shown in Figure 7D and (C). Lower: samples from two patients who presented CR clinically. Each image represents a different sample. Dashed lines and arrows:
CRATERs. All samples are labeled with the organ from which they were extracted.
(E) Four representative samples out of nine analyzed samples of metastases taken post treatment from patients who presented clinically a progressing disease.
Each image represents a different sample. Yellow arrows: CD8^+^ T cells infiltrating the tumor mass. All samples are labeled with the organ from which they were extracted.
(F) Regionality in CRATER formation in two samples of patients who responded to ICB. Gray lines: PMB. Red lines: CRATERs. Yellow arrows: foci of CRATERs within the tumor mass. Patient A presented CR. Patient B presented SD.
(G) CRATER density in untreated tumors, correlated with a later response to treatment (*n* = responders-4, non-responders-10. Whiskers represent 1.5× the interquartile range. *t* test. Non-significant).
(H) mIF of untreated human cutaneous melanoma stained for apoptotic marker-activated Cas3, pre-(upper) and post- (lower) treatment. Images present CRATER areas in which CD8^+^ T cells are not contacting (image #1) or contacting (image #2) apoptotic cells. Red lines: CRATERs. Yellow lines: PMB. White arrows: activated Cas3^+^(apoptotic) cells.
(I) Percentage of CRATERs containing CD8^+^ T cells directly interacting with activated Cas3^+^ cells, from total CRATERs in matched pre- and posttreatment biopsies of patients, each for the same patient. Each line represents a patient.
(J) (1,2) Two representative images of non-small cell lung cancer (NSCLC) not yet forming a lobular mass, in which CD8^+^ T cells are mostly scattered at the stromal area; single cells engage tumor cells. (3,4) Representative images of developed tumors. Multiple CD8^+^ T cells at the stromal area. CD8^+^ T cell clusters appear in pockets extending into the cytokeratin (CK)^+^ lung cancer tumor mass. White arrows at the right image: CRATERs. Areas marked by white rectangles are enlarged at the bottom of each image, where CRATER areas are marked by white dashed lines. Representatives of 15 samples reviewed.
(K) Images of vascularized, stromal tissue bordering the malignant cell nest in developed NSCLC tumors. Images present areas with and without CRATERs. Areas in the rectangles are enlarged at the bottom. (1) The boundaries of the malignant cell nest are smooth, without CRATERs. (2) Presence of a CRATER (white dashed line) that lacks collagen and harbors CD8^+^ and CD163^+^ cells.
(L) Another example of a CRATER (white dashed line) lacking collagen and harboring CD8^+^ and CD163^+^ cells in NSCLC.

8

9

10

11

12

13

14

SUPPLEMENTAL INFORMATION

Supplemental information can be found online at https://doi.org/10.1016/j.cell.2025.09.021.

## Figures and Tables

**Table:** KEY RESOURCES TABLE

REAGENT or RESOURCE	SOURCE	IDENTIFIER

Antibodies		

Anti- collagen (clone: EPR7785)	Abcam	RRID:AB_2861258; Cat: ab138492
Anti-SOX10 (clone EP268)	Cell Marque	RRID:AB_2941085; Cat:AC-0237A
Anti-CD31 (clone ab28364)	Abcam	RRID:AB_726362; ab28364
Anti-CD8 (clone C8/144B)	Dako	RRID:AB_2075537c; Cat #: M710301-2
Anti-S100a (clone EPR525)	Abcam	Cat #: ab133519
Anti-CD163 (clone 10D6)	Leica	RRID:AB_2920861; Cat #: CD163-L-CE
Anti-CD105 (clone EPR19911-220)	Abcam	RRID:AB_3662768; Cat #: Ab252345
Cleaved Caspase-3 (Asp175) (5A1E)	CST	RRID:AB_2070042; Cat #: 9664
Anti mouse β2-microglobulin (clone A16041A)	Biolegend	RRID:AB_2721309; Cat #:154506
Anti CD8α (clone CAL38)	Abcam	RRID:AB_2864723; Cat #: ab237723
Rhodamine (TRITC) AffiniPure^™^ Goat Anti-Rabbit antibody	Jackson ImmunoResearch	RRID:AB_2337926; Cat #: 111-025-003
anti-mouse plus anti-rabbit Opal Polymer Horseradish Peroxidase	Akoya Biosciences	RRID:AB_28909; Cat #: ARH1001EA
Cytokeratin (clone AE1/AE3)	Agilent Dako	RRID:AB_28920; Cat #: GA053

Bacterial and virus strains		

One Shot^™^ TOP10 Chemically Competent *E. coli*	Invitrogen	Cat #: C404003

Biological samples		

Human melanoma and NSCLC blocks	DFCI/BWH	NA

Chemicals, peptides, and recombinant proteins		

TRIzol^™^ Reagent	Invitrogen	Cat #: 15596026
Penicillin G Sodium	Sigma Aldrich	Cat #: P3032
Streptomycin Sulfate	Sigma Aldrich	Cat #: S6501
Amphothericin B	Santa Cruz Biotechnology	Cat #: SC-202462B
Keflex/Cefalexin	Fish Mox Fish Flex	N/A
Prazipro/Praziquantel	Hikari Usa	Cat #: AHK73254
Ovalbumin Alexa Flour 594 conjugate	Invitrogen	Cat #: 11559176
HBSS medium	Gibco	Cat #: 14025092
Fetal calf serum	Atlanta Biologicals	Cat #: S11150
SYBR Green Real-Time PCR Master mix	ThermoFisher Scientific	Cat #: A25741
RNase inhibitor	Takara Bio	Cat #: 2313A
dNTP	New England Biolabs (NEB)	NA
dithiothreitol	Bioworld	Cat #: 40420001-1
Betaine	Sigma	Cat #: B0300-5VL
MgCl_2_	Sigma	Cat #: M1028-10X1ML
Tricaine-S	Syndel	Cat #: NC0342409
Triton X-100	Sigma-Aldrich	Cat #: 93443
Sytox Red	Thermo Scientific	Cat #: S34859
KAPA HiFi HotStart ReadyMix	Kapa Biosystems	Cat #: KK2602
Lambda Exonuclease	NEB	Cat #: M0262L
AMPure XP bead	Beckman Coulter	Cat #: A63882
EB	Qiagen	Cat #: 1014609
Liberase	Sigma	Cat #: 05401119001
Tissue-Tek O.C.T Compound	Sakura	Cat#4583
CpG ODN 2007	Invivogen	Cat #: tlr-2007
SB431542	Sigma Alderich	Cat #: 301836-41-9
4% PFA in PBS	Boston BioProducts	Cat #: BM-155
Hoecsht 33342	Biotium	Cat #: 40046
Fluoromount Aqueous Mounting media	Sigma	Cat #: F4680
Dynabeads^™^ Mouse T-Activator CD3/CD28 for T-Cell Expansion and Activation	ThermoFisher scientific	Cat #: 11456D
Spectral DAPI (4’, 6-diamidino-2-phenylindole)	Akoya Biosystems	Cat #: FP1490
BOND DeWax Solution	Leica Biosystems	Cat #: AR9590
BOND Epitope Retrieval Solution 1 (pH 6)	Leica Biosystem	Cat #: AR9961, AR9640
Prolong Diamond Anti-fade mounting medium	Life Technologies	Cat #: P36965

Critical commercial assays		

SMART-Seq^®^ Ultra^™^ low Input RNA kit for sequencing	Clontech	Cat. #s: 634888, 634889, 634890, 634891, 634892, 634893, and 634894
Nextera XT DNA library preparation kit	Illumina	Cat #: 15028252
Nextera XT index kit	Illumina	NA
MinElute PCR purification kit	Qiagen	Cat #: 28004
SMARTScribe Reverse Transcriptase	Takara Bio	Cat #: 639538
Quant-iT dsDNA High Sensitivity kit	Thermo Fisher	Cat #: Q33120
DNeasy blood and tissue kit	Qiagen	Cat #: 69506
Phusion^®^ High-Fidelity PCR Master Mix with HF Buffer	NEB	Cat #: M0530
mMESSAGE mMACHINETM SP6 Transcription Kit	Invitrogen	Cat #: AM1340
RNAScope^™^ Fluorescent multiplex assay	ACDBio	NA
Click-iT^™^ Plus TUNEL Assay Kit	Fisher Scientific	Cat #: C10619
Mouse CD8^+^ T Cell Isolation Kit	StemCell Technologies	Cat #: 19853
Live and Dead Cell Assay	Abcam	Cat #: ab115347
200- or 300-cycle kit	Illumina	Cat #: 20012861 and 20012860
pENTR^™^ 5’-TOPO^™^ TA Cloning^™^ Kit	Invitrogen	Cat #: K59120

Deposited data		

RNA-Seq and ATAC-Seq zebrafish data	This paper	NCBI Gene Expression Omnibus (GEO): GSE213286

Experimental models: Cell lines		

UV2 mouse melanoma cell line	Wucherpfennig lab	PMID: 33597266

Experimental models: Organisms/strains		

Tg(*cd8α*:EGFP)	This paper	NA
Tg(*lck*:mCherry)	This paper	NA
Tg(*cd8a*:EGFP;lck:mCherry)	This paper	NA
Tg(*cd8a*:EGFP; BRAF^V600E^/p53^null^/nacre^null^)	This paper	NA
Tg(*flk*:EGFP)	Zon lab	NA
Tg(*fli1ep*:dsred)	Zon lab	NA
Tg(fli1ep:dsRedex, flk1:EGFP) zebrafish	Zon lab	NA
Tg(*cd8a*:EGFP;prkdc^D3612fs^ ) zebrafish (prkdc−/−)	Langenau lab	NA
Jak3^P369fs^ (Jak^(−/−)^)	Langenau lab	NA
Prkdc−/− zebrafish	Langenau lab	NA
Rag^(−/−)^ mice	Jackson laboratories	RRID:IMSR_JAX:002216

Oligonucleotides		

gRNA targeting zebrafish tyrosinase:	Zon lab (J. Ablain)	5’-GGACTGGAGGACTTCTGGGG-3’
gRNA targeting zebrafish p53:	Zon lab (J. Ablain)	5’-GGTGGGAGAGTGGATGGCTG-3’
gRNA targeting zebrafish b2m:	Zon lab (C. Pessoa-Rodriguez)	5’- TAAATCCAAACCGGGCAGCG-3’
Primers for detecting b2m deletion:	This paper	FW: 5’-CATCTTCGCAATACCTTAGGCT-3’; RV: 5’-TCAGCTAAGGTAAGTGAACAGGCCTTAATTTGGAC-3’
cd8a promoter primers:	This paper	First amplification: FW: 5’-CTGTATCTCTGTGTGTGCGT-3’ Rv: 5’-GTGGCACCCTTTAACATGATCG-3’; Second amplification: FW: 5’-TGAGGTTCGTCAGCAGACTTG-3’ Rv: 5’- CCAGTTTCCAGCACAAGCATTC-3’
BRAF/p53/Nacre genotyping primers:	White et al.^7^	WT BRAF: Fw: 5’-TGCTCTTGACCTCAGACTGG-3’; Rv: 5’-CCTCAATAAACAC CCTACGG-3’; BRAF V600E Fw: 5’-GAGGCTTTGTCGAATCGGACCGGTG-3’; Rv: 5’-TTGAACAGAGCCTGGCCCGGCT-3’ p53; Fv: 5’-TGTGTCTGTCCATCTGTTTAACAGTCA-3’; Rv: 5’-GATAGCCTAGTGCGAGCACACTCTT-3’
oligo-dT primer	IDT	5′-AAGCAGTGGTATCAACGCAGAGTACT30VN-3′
Prkdc−/− genotyping primers	Moore et al.^11^	Fw: 5’- CAGGACTGGTGGGATGAGGT −3’; Rv: 5’- CATAGCATATCAGAATTTTGGGCTT-3’
Jak3−/− genotyping primers	Moore et al.^11^	Fw:5’- TTCAAACCCTGACAGACGCCCTTT-3’; Rv: 5’- AGGAATGGACTAGGATGTGTCCCA-3’

Recombinant DNA		

MAZARATI Vector	Zon lab (J. Ablain)	NA
MiniCoopR Vector	Zon lab	NA
pDestTol2CG2	Zon lab	NA
p5-ENTER-lck 5.5 plasmid	Langeanu lab	NA
p-ENTER-zf mCherry vector	Zon lab	NA
p-ENTER-EGFP vector	Zon lab	NA
P3-ENTER-polyA vector	Zon lab	NA
p5-ENTER-zfCD8a vector	This paper	NA

Software and algorithms		

Imaris	Oxford Instruments	NA
Arivis Vision4D 3.4-3.6	ZEISS	NA
FACSDiva software	Becton Dickinson	NA
QuPath	Bankhead et al.^48^	NA
Tophat 2.0.11	Kim et al.^49^	NA
Cufflinks 2.2.1	Trapnell et al.^50,51^	NA
Cutadapt software	Martin^52^	NA
FastQC sortware	(http://www.bioinformatics.babraham.ac.uk/projects/fastqc/)	NA
Bowtie2 (version 2.2.1)	Langmead and Salzberg^53^	NA
MACS2 version 2.1.0 peak finding algorithm	Zhang et al.^54^	NA
ClipChamp software	Microsoft	NA
STAR 2.7.0 Spliced Transcripts Alignment tool	Dobin et al.^55^	NA
R v4.0.4 software	R Core Team ^56^	NA
Seurat v4.0.2 and Seurat scRNA-seq integration SCTransform	Hao et al.^57^ Stuart et al.^58^	NA
CrispRVariants	Lindsay et al.^59^	NA
Density-Based Spatial Clustering of Applications with Noise (DBSCAN)	Ester et al.^60^	NA
Cellpose with the TissueNet model	Stringer et al.^61^	NA
Rolling ball algorithm	NA	NA
Scanpy python package	Wolf et al.^62^	NA
UMAP	Becht et al.^63^	NA
Leiden clustering	Traag et al.^64^	NA
Slide-Seq pipeline tool	https://github.com/MacoskoLab/slideseq-tools	NA
Leica LAS X software	Leica	NA
Biorender	https://app.biorender.com/	NA
Photoshop software	Adobe	NA

Other		

10 μl 1701SN 33’/0.6”/2 Hamilton syringe (intra tumoral injections)	Hamilton	NA
Superfrost histological slides	Fisher scientific	NA
inverted AxioObserver with LSM 900 scanhead microscope	ZEISS	NA
Axio Examiner upright microscope	ZEISS	NA
BOND RX fully automated stainer	Leica Biosystems	NA
Vectra Polaris multispectral imaging platform	Vectra Polaris, Akoya Biosciences	NA
tissue-coupled VivaScope 1500 or handheld VivaScope 3000	Caliber I.D	NA
FACs Aria III	Becton Dickinson	NA
Illumina Hiseq 2500 platform	Illumina	NA
Agilent Bioanalyzer	Agilent	NA
LSM 880 microscope	ZEISS	NA
ZEISS LSM 980 scanhead coupled to an Axio Examiner upright microscope	ZEISS	NA
NovaSeq 6000 Sequencing System	Illumina	NA
Inverted AxioObserver with LSM 900 scanhead microscope	ZEISS	NA
Leica M165 FC fluorescent microscope	Leica	NA
Axio Examiner upright microscope	ZEISS	NA
KNF N86 KTP vacuum pump	Cole Parmer	NA
Ismatec REGLO Digital 4-channel 12 Roller variable speed pump	Cole Parmer	NA
SH-27B in-line heater and controlled by TC-324 heater controller	Harvard Apparatus	NA
ProSense digital pressure sensor	AutomationDirect	NA
SpectraMax i3x microplate reader	Molecular Devices	NA
Vectra Polaris multispectral imaging platform	Vectra Polaris, Akoya Biosciences	NA

## Highlights

- CRATERs are pockets of elevated antigen presentation on the melanoma surface
- CD8^+^ T cells preferentially engage with tumor cells within CRATERs
- During immune stimulation, CRATERs expand and become the major site for tumor killing
- CRATERs expansion correlates with patients’ clinical benefit following immunotherapy
