# Supplementary figures and images for: CRATER tumor niches facilitate CD8+ T cell engagement and correspond with immunotherapy success
Source: Cell. Author manuscript; Available in PMC 2025 Nov 12. (PMC12604482; doi:10.1016/j.cell.2025.09.021)

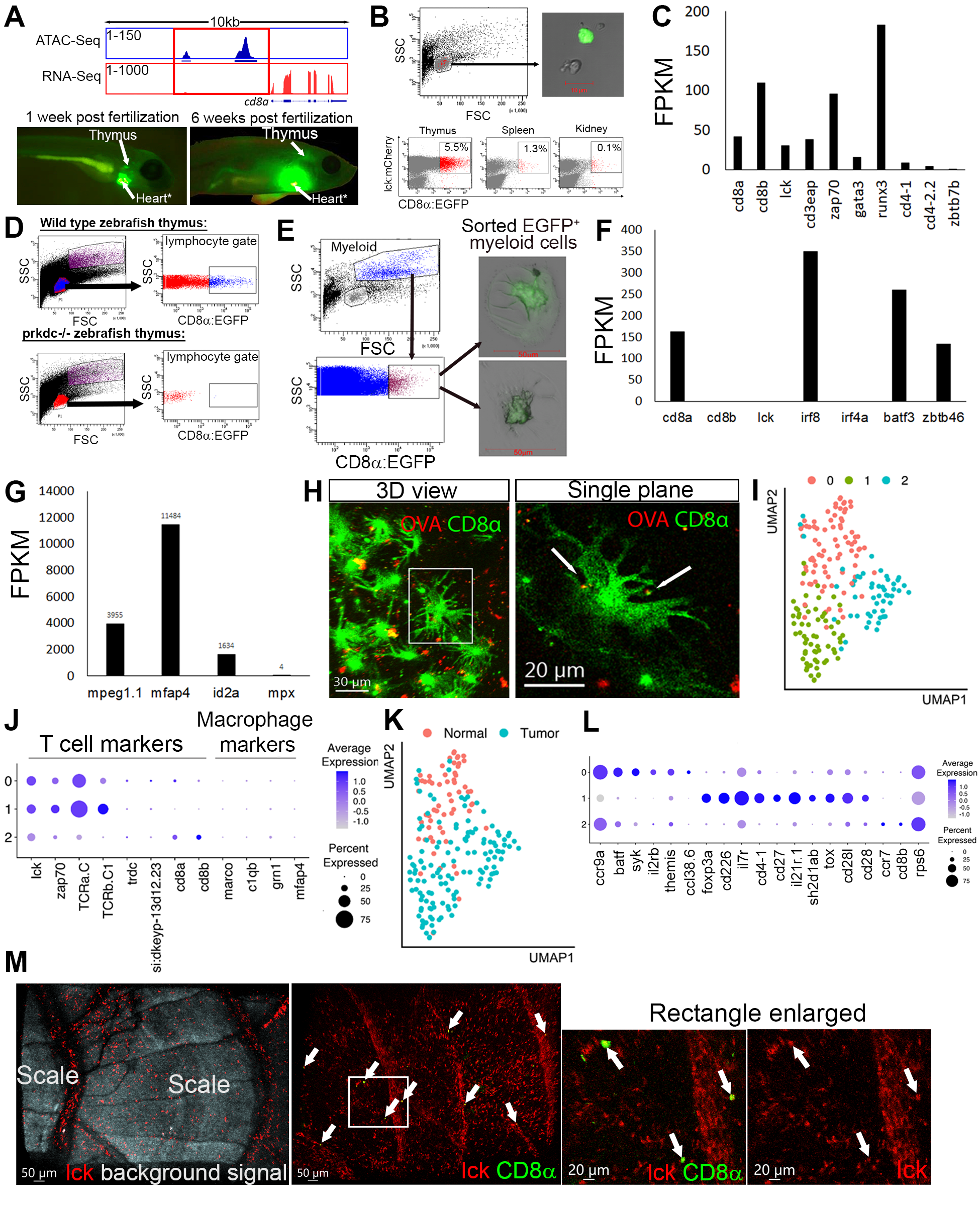

Supplement: 1 — (A) Upper: cd8a gene area in ATAC-seq and bulk RNA-seq analyses of sorted lck+ lymphocytes from zebrafish thymi. Rectangle: area used as cd8a promoter. Lower: fluorescent images of zebrafish at 1 and 6 weeks post fertilization. Arrow: EGFP+ zebrafish thymi. *EGFP expression in the heart is irrelevant for cd8a expression. It results from the backbone plasmid used to select successful transgenes. (B) Flow cytometry analysis of Tg(cd8α:EGFP; lck:mCherry) zebrafish thymus-derived cells. Upper: FSCC plot of zebrafish thymus cells. Red population: EGFP+gated population appears at the lymphoid gate. Upper right image: EGFP+ lymphocytes sorted onto slides showing a bright EGFP+ cell of ∼10 μm in diameter. Lower: lymphoid-gated flow cytometry plots of thymus, spleen, and kidney cells, showing CD8α:EGFP+/lck+ cells. (C) T cell gene expression in bulk RNA-seq of EGFP+ thymus-derived sorted cells from 8-week-old (juvenile) Tg(cd8α:EGFP) zebrafish. (D) Flow cytometry plots of thymi cells collected from WT and prkdc(−/−) Tg(cd8a:EGFP ) adult zebrafish. (E) Flow cytometry plots of melanoma-derived cells showing CD8α:EGFP+ cells in the myeloid gate. Right: cd8a:EGFP myeloid cells sorted on a slide. (F and G) Bulk RNA-seq of EGFP+ myeloid cells sorted from melanoma tumors showing expression of genes associated with (F) CD8+ T and dendritic cells and (G) myeloid cells. (H) In vivo imaging of the thymic region of Tg(cd8α:EGFP) zebrafish injected intraperitoneally (i.p.) with ovalbumin-AlexaFluor 594. Left: 3D view of CD8α:EGFP dendritic cells. Right: rectangle enlarged, presenting a single plane (2 um slice), where OVA fragment is presented on the DC dendrites. (I) Single-cell RNA-seq of sorted lck+ T cells, using Tg(lck:mCherry) zebrafish melanoma and normal skin, exhibit 3 clusters (a macrophage cluster, which was a contamination, was omitted from the dataset). (J) Expression of select genes classifying T cell clusters and presenting the lack of expression of macrophage markers in t [file NIHMS2117968-supplement-1.tif]

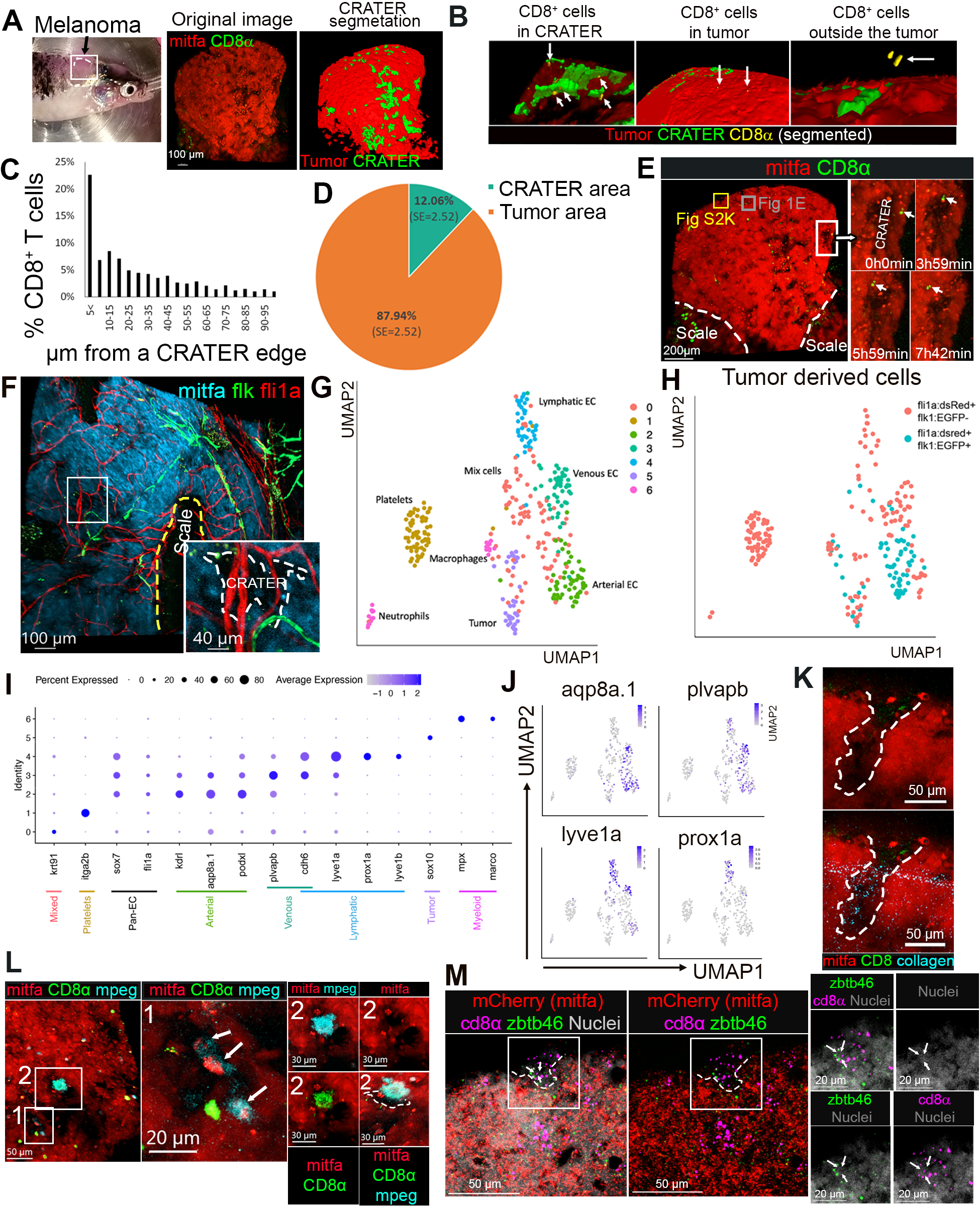

Supplement: 2 — (A) Left: zebrafish with an mCherry+ melanoma protruding behind its head (the fish is intubated for long-term time-lapse imaging). White dashed line marks the upper border of the fluorescent image (mirror image). Middle: the fluorescent image of the area marked with the white rectangle on the left image. Right: outcome of tumor and CRATER segmentation, as described in the STAR Methods, of the image in the middle. The areas lacking signal and corresponding to CRATERs are now segmented objects marked in green. (B) Examples of CD8+ cells quantified as “in CRATER” and “in tumor.” Yellow arrows mark CD8+ cells. The cells were quantified when directly connecting the segmented areas per segmented surface (mm2). Some CD8+ cells were found in the epithelial layer covering the tumor, i.e., “outside” the tumor (upper image), and were not included in the analyses of this study, unless indicated otherwise. (C) CD8+ cell distribution by distance from CRATERs, showing fewer CD8+ cells as the distance from CRATERs increases (n = 6 fish, calculated for 1,200 cells). (D) CRATER area constitutes about 12% of the tumor surface in untreated tumors (n fish = 5, data are mean + SE). (E) Snapshots of long-term live imaging. Left: low magnification of the imaged tumor. Yellow rectangle is enlarged in (K), gray rectangle is enlarged in Figure 1E. The white rectangle to the right is enlarged in the right panel. CD8+ T cells (white arrows) linger in a CRATER for nearly 6 h before moving out. Time is hours: minutes. (F) 3D image of a mitfa:BFP tumor in Tg(flk:GFP; fli1a:dsRed) zebrafish. Dashed yellow line outlines a scale protruding from the tumor mass, in the lowmagnification image. A CRATER area is enlarged at the low right corner, showing proximity to fli1a+/flk− and flk+ blood vessels. White dashed line: CRATER. (G) Single-cell RNA-seq analysis of sorted fli1a+/flk1+ and fli1a+/flk1− cells from zebrafish melanoma and normal skin using the SORT-seq technology exhibits 7 clusters. (H) Endoth [file NIHMS2117968-supplement-2.tif]

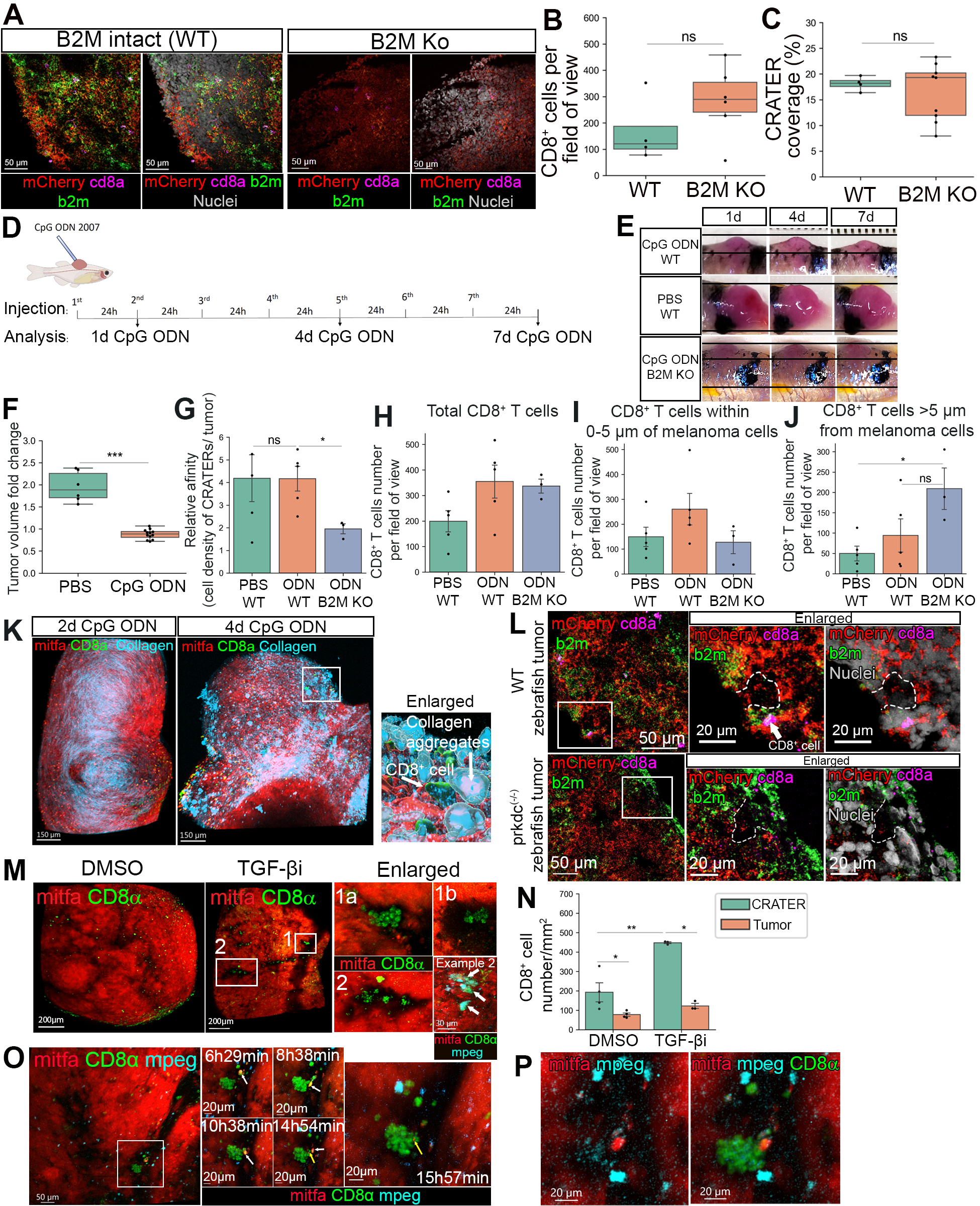

Supplement: 3 — (A) RNAscope assay detecting mRNA of mCherry, cd8a, and b2m RNAs in B2M intact and B2M KO zebrafish tumors. Representative of 5 FOV, 2 fish for each group (WT or B2M KO). (B and C) (B) CD8+ cell number per field of view and (C) CRATER coverage (% of the tumor surface) in tumors of intact (WT) and melanocyte-specific B2Mdepleted melanomas. (For both graphs: dots represent fish. n = 4 control fish, 6 FOV, 5 B2M-depleted fish, 8 FOV. t test. p value for (B) = 0.34. p value for (C) = 0.12. Whiskers represent 1.5× the interquartile range). (D) A diagram of CpG ODN treatment applied to zebrafish melanoma. Created using Biorender.com. (E) Representative images of melanoma tumors injected daily with CpG ODN or PBS for 1, 4, and 7 days, each 24 h post last injection. Upper: WT tumor injected with CpG ODN; middle: WT tumor injected with PBS; lower: B2M KO tumor injected with CpG ODN. (F) Fold change of tumor volume following seven daily injections of vehicle (PBS) or CpG ODN intratumorally into zebrafish. (Dots represent fish. n = PBS-6 fish, CpG ODN-12 fish. Whiskers represent 1.5× the interquartile range. t test. p value = 0.0001). (G–J) (G) Relative affinity of CD8+ T cells for CRATERs (CD8+ T cell density in CRATERs/tumor), (H) CD8+ T cells numbers per field of view, (I) numbers per field of view of CD8+ T cells near or contacting tumor (<5 μmaway from tumor), and (J) numbers per field of view of CD8+ T cells away from or not contacting tumor (>5 μm away from tumor), all in PBS-, CpG ODN-, and tumor-specific B2M KO, CpG ODN-treated melanomas (G–J calculated for samples shown in Figures 3A and 3B: dots represent fish. n = PBS-5, CpG ODN-5, B2M KO CpG ODN-4 fish. 2–3 FOV per fish, data are mean ± SE. t test. *p value = 0.01). (K) 3D image using second harmonic generation (SHG) microscopy to visualize collagen of CpG ODN-injected tumors in Tg(cd8α:EGFP) zebrafish (representative of 2 CpG ODN 2d- and 4 CpG ODN 4d-injected fish). CD8+ cell infiltration after 4 daily injections [file NIHMS2117968-supplement-3.tif]

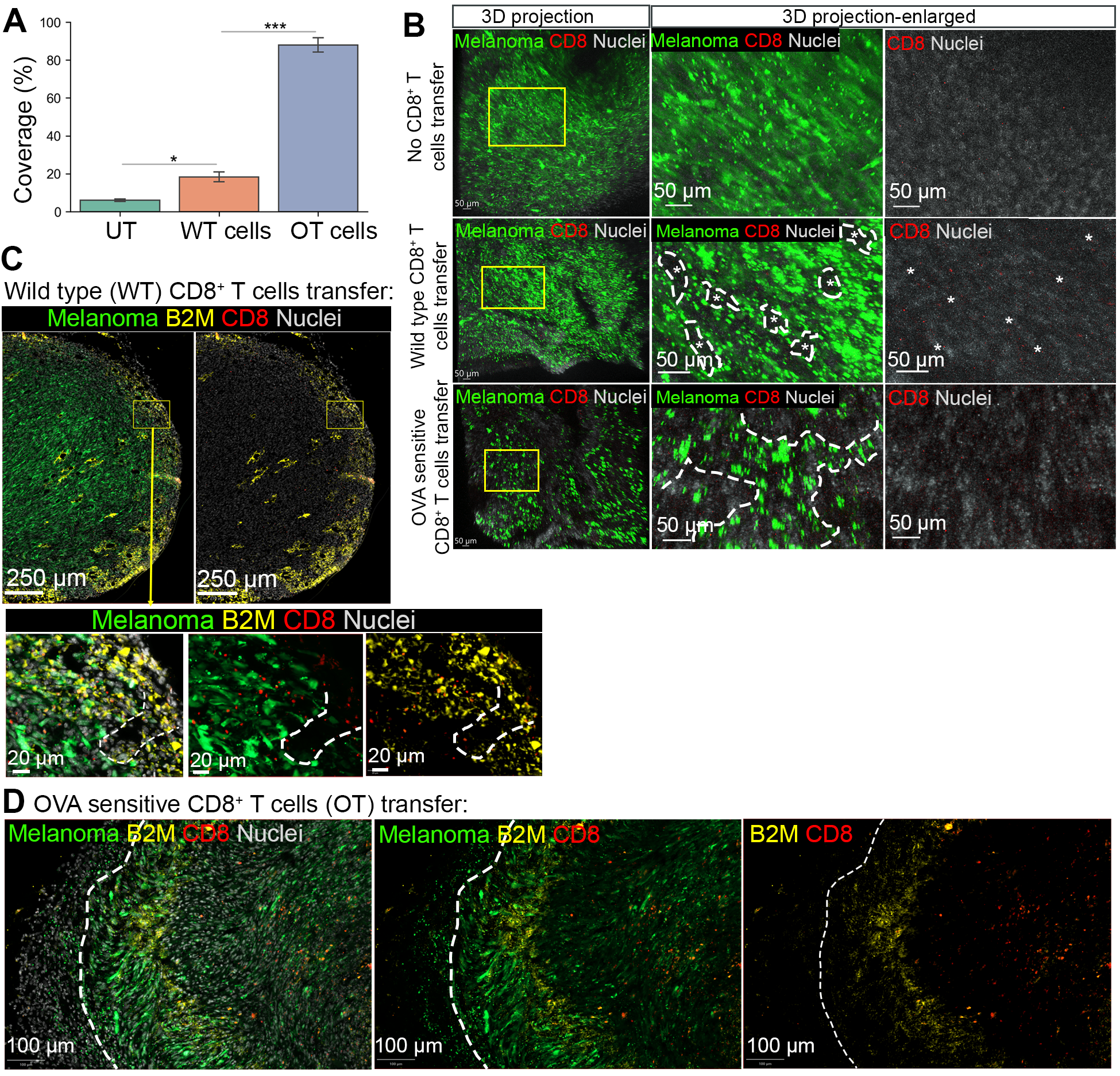

Supplement: 4 — (A) Percent of tumor surface lacking tumor cells, measured through 3D projection of whole-mount staining of a lobe resected from OVA-expressing tumors in mice undergoing no CD8+ T cell transfer (UT), activated, non-OVA-sensitive, wild-type CD8+ T cells (WT), or OVA-sensitive CD8+ T cells (OT). (n mice: UT = 5, WT = 5, OT = 5 mean ± SE. t test *p value = 0.03, **p value = 0.0001). (B)Representative images of 3D projections of tumor lobes described in (A). Left column: broad-field view. Right images: areas marked by rectangles are enlarged and shown with and without the melanoma marker. Asterisks and dashed lines: areas lacking tumor cells, resembling CRATERs in WT CD8+ T celltransferred tumors. (C) Immunofluorescence staining of 2D sections of tumors following WT CD8+ T cell transfer. Upper: low magnification, presenting the widespread expression of B2M on the tumor surface, shown with and without melanoma marker. Lower: rectangle enlarged. An area lacking melanoma cells that resembles a CRATER is marked by dashed white line. B2M staining as well as CD8+ T cells can be found within the CRATERiform area and its surroundings. (D) A representative image of immunofluorescence staining of 2D sections of tumors following OVA-sensitive CD8+ T cell transfer. The same area is shown for different marker combinations. Dashed white line: the tumor surface. Widespread tumor attrition and B2M expression is present. CD8+ T cells are found within the tumor mass rather than at the tumor surface. [file NIHMS2117968-supplement-4.tif]

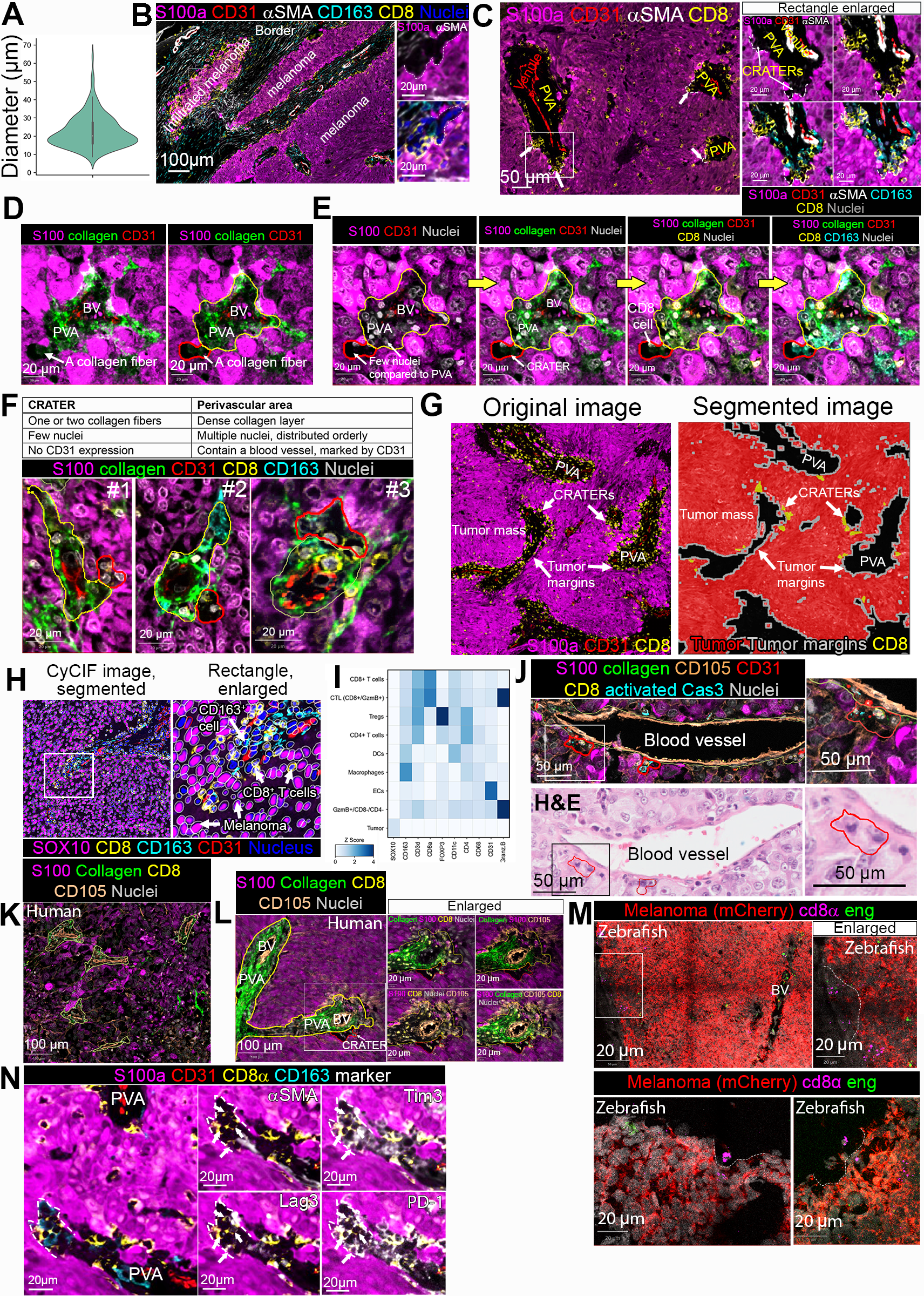

Supplement: 5 — (A) Violin plot depicting CRATERs diameter distribution in human melanoma (measured for n = 714 CRATERs, 2 samples). (B and C) CyCIF images of (B) S100+ melanoma tissue contacting the stromal layer at the outer border of the tumor and (C) at the perivascular area. Left: low magnification. Arrows and dashed lines mark examples of CRATERs. Right: rectangle area, enlarged, presenting CRATERs containing CD8+ T cells and CD163+cells. Steps for CRATER identification, from left to right, all show the same CRATER. (D) Perivascular area (PVA) is detected by CD31+ blood vessel (BV) surrounded by a layer of collagen fibers. This area will not contain S100+ melanoma cells. Its boundaries, i.e., perivascular-melanocytic boundaries (PMB), are marked by a yellow line. A 20 μm protrusion without melanoma cells and dense collagen is marked by a red line. It contains one fiber of collagen. (E) Left: perivascular area (PVA), whose boundaries are marked by a yellow line, has dense nuclei content, whereas the area marked by the red line has very few nuclei. Thus, the red marked area is designated as a CRATER. The CRATER contains a CD8+ cell (middle) and CD163+ cell (right). (F) Top: a table distinguishing PVA indentation and CRATER areas. Bottom: three additional examples of CRATERs. (G) An example of segmentation applied on a CyCIF image, as done in this study. The image on the left is segmented on the right, highlighting the CRATER areas (yellow), tumor margins, i.e., PMBs (gray), and tumor mass (red). See STAR Methods for a detailed description of the analysis. (H) Example of cell segmentation of a human CyCIF sample of primary human melanoma as analyzed in this study. Right: rectangle area, enlarged, to show the individual cells. (I) Cell segmentation was followed by cell clustering and identification of cell populations stained in the sample: signal intensity heatmap of markers read in the CyCIF samples analysis, identifying cell populations. (J) mIF followed by washing and stainin [file NIHMS2117968-supplement-5.tif]

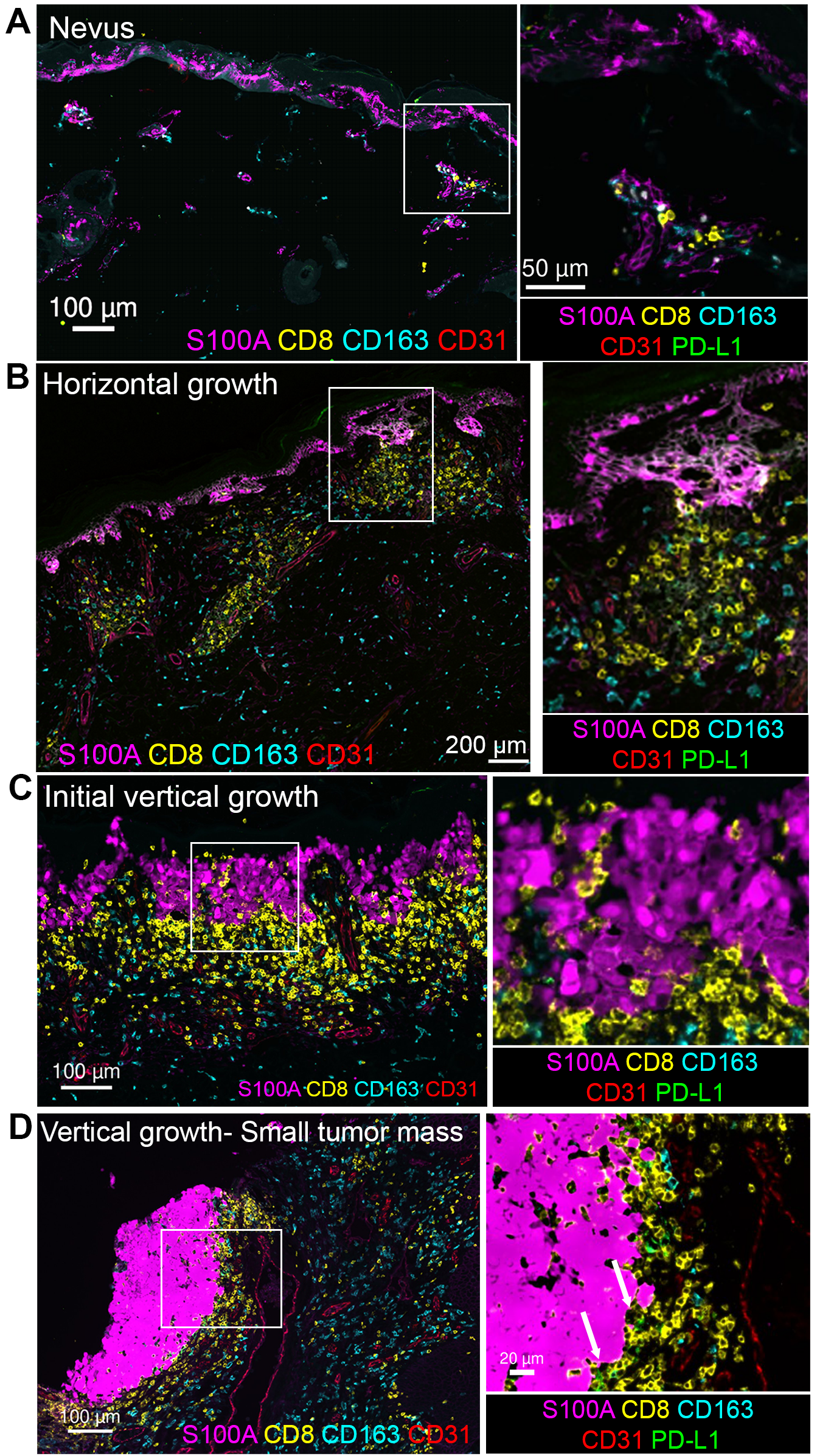

Supplement: 6 — (A) Nevus. (B) Horizontal growth, with CD8+ T cells aggregating around blood vessels. (C) The tumor thickens, and multiple CD8+ T cells contact its margins. PD-L1 negative indentations are seen at the tumor margins. (D) Vertical growth area forming a small tumor mass in melanoma at the vertical growth phase. CRATERs (marked by white arrows) appear at the melanoma border containing CD8+ T cells and PD-L1 expressing CD163+ DCs. Images are representatives of the following reviewed samples: superficial radial phase: n = 15. Vertical growth: n = 3. [file NIHMS2117968-supplement-6.tif]

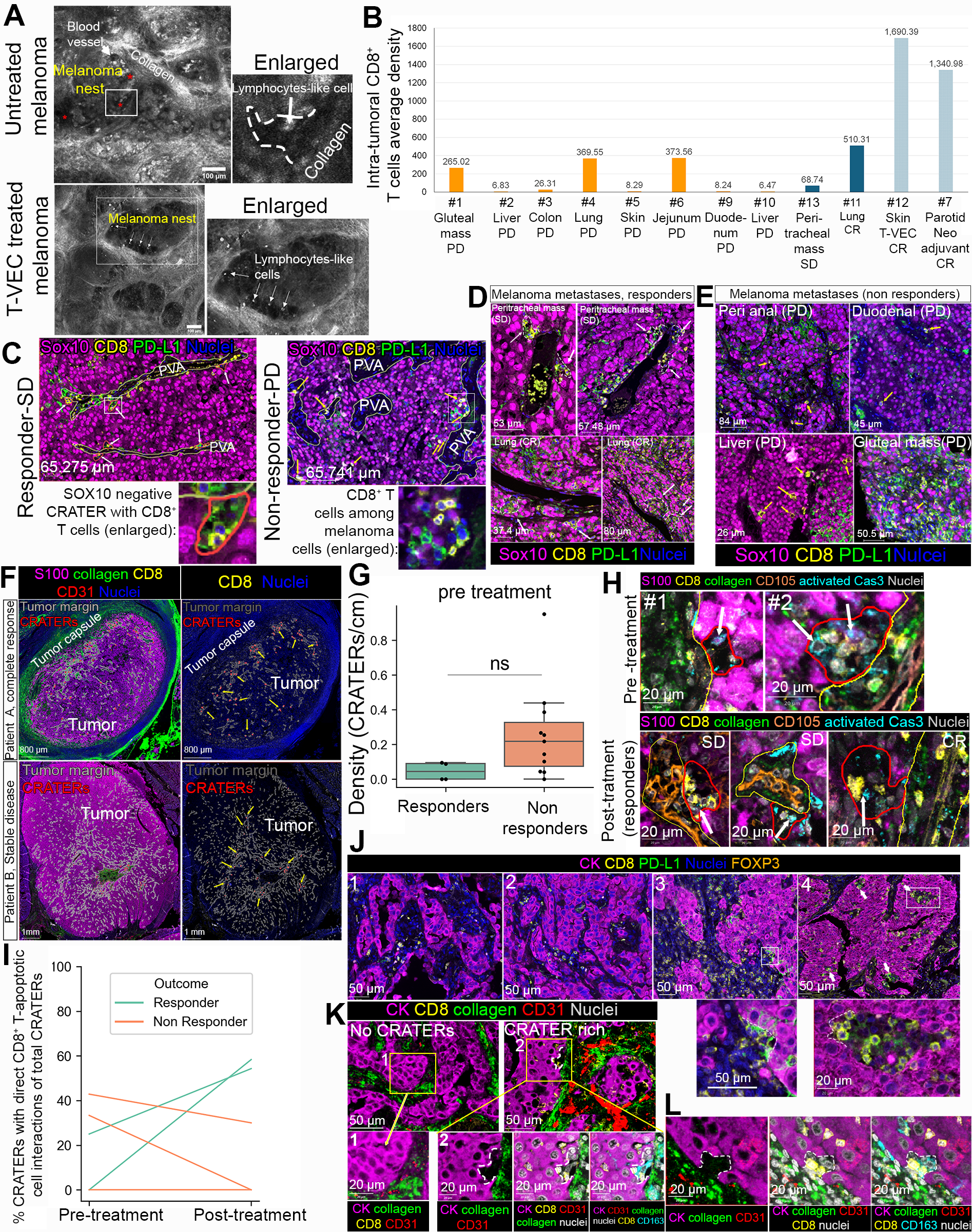

Supplement: 7 — (A) In vivo 3D confocal view of melanoma nests in patients using RCM. Upper: pockets akin to CRATERs can be found at the edge of a melanoma nest (enlarged). Lower: melanoma nests in a patient treated with T-VEC. The nests are emptying of cells, and large pockets akin to CRATERs appear together with lymphocytelike cells (enlarged). White arrows indicate lymphocyte-like cells, as determined by pathologists reviewing the images (see STAR Methods). (B) Intra-tumoral CD8+ T cell density value, measured at the pathology department of BWH, for each of the posttreatment samples analyzed. Graph depicts average intratumoral CD8+ T cell number per mm2 for at least 6 regions of interest per sample. (C) Representative images of metastases resected post treatment. Left: durable response (SD). Yellow lines: PMB, arrows: CRATERs. Right: PD. Yellow arrows: CD8+ T cells entering the tumor. At the bottom of each image: rectangle area, enlarged. In durable response, CD8+ T cells aggregate in CRATERs (red line). In PD, most CD8+ T cells are found as singles or doublets, spread among melanoma cells. (D) Additional representative images from durable response to ICB treatment. Upper: two additional areas from the SD sample: the same sample shown in Figure 7D and (C). Lower: samples from two patients who presented CR clinically. Each image represents a different sample. Dashed lines and arrows: CRATERs. All samples are labeled with the organ from which they were extracted. (E) Four representative samples out of nine analyzed samples of metastases taken post treatment from patients who presented clinically a progressing disease. Each image represents a different sample. Yellow arrows: CD8+ T cells infiltrating the tumor mass. All samples are labeled with the organ from which they were extracted. (F) Regionality in CRATER formation in two samples of patients who responded to ICB. Gray lines: PMB. Red lines: CRATERs. Yellow arrows: foci of CRATERs within the tumor mass. Patient A presented CR. [file NIHMS2117968-supplement-7.tif]
