# Supplementary material for: CRATER tumor niches facilitate CD8+ T cell engagement and correspond with immunotherapy success
Source: Cell. Author manuscript; Available in PMC 2025 Nov 12. (PMC12604482; doi:10.1016/j.cell.2025.09.021)
Supplement: 8 [file NIHMS2117968-supplement-8.pdf]

	Sex	Age	Melanoma type	Treatment length	Drug	Treatment setting	Time to biopsy	outcome of treatment	Time to clinical outcome	Location	Designation in paper	Lesion type
1 post	Male	60	Cutaneous	2 months	Ipilimumab + Nivolumab	Metastatic	3 months	PD	3 months	gluteal mass	Non-Responder	Metastasis
2 post	Male	52	Cutaneous	7 months	Dabrafenib + Trametinib + Pembrolizumab	Metastatic	7 months	SD mixed response	7 months	liver	Non-Responder	Metastasis
3 post	Male	84	Mucosal	6 months	Pembrolizumab	Adjuvant	4 months	PD	6 months	Anal colon	Non-Responder	Metastasis
4 post	Female	59	Cutaneous	8 months	Nivolumab	Metastatic	8 months	PD	5 months	lung	Non-Responder	Metastasis
5 post	Male	64	Cutaneous	5 months	Ipilimumab + Nivolumab (3m), Nivolumab (2m)	Metastatic	7 months	PD	6 months	Skin	Non-Responder	Metastasis
6 post	Female	56	Cutaneous (Acral Lentiginous)	18 months	Ipilimumab	Adjuvant	22 months	PD	27 months	jejunum	Non-Responder	Metastasis
7 post	Male	82	Cutaneous	10 months	Nivolumab	Neoadjuvant	3 months	CR	20 months	right parotid	Responder	Metastasis
8 post	Male	77	Mucosal (anal)	1 month	Pembrolizumab	Adjuvant	1m	PD	5 months	Anal (Mucosal)	Non-Responder	Metastasis
9 post	Female	56	Cutaneous	5 months	Nivolumab	Adjuvant	5 months	PD	2 months	Duodenum	Non-Responder	Metastasis
10 post	Female	65	Mucosal (Nasal)	2 months	Nivolumab	Adjuvant	2 months	PD	2 months	liver	Non-Responder	Metastasis
11 post	Female	68	Cutaneous	40 months	Pembrolizumab	Metastatic	30 months	CR	47 months	Lung	Responder	Metastasis
12 post	Male	72	Cutaneous	2 months (P), 7 months pause, 2 months (T)	Pembrolizumab (2m) then T-VEC (2 months)	Adjuvant	12 months (after TVEC)	CR	17 months	Skin	Non-Responder (to ICB)	Metastasis
13 post	Female	33	Cutaneous	9 months	Ipilimumab + Nivolumab + Bevacizumab	Metastatic	11 months	SD	6 months	Sub peritracheal mass	Responder	Metastasis
A pre	Male	60	Cutaneous	-	Nivolumab	Adjuvant	NA	SD	-	skin	Non-Responder	Metastasis
B pre	Female	59	Cutaneous	-	Nivolumab	Adjuvant	NA	PD	-	Skin	Non-Responder	Metastasis
C pre	Male	85	Cutaneous	-	Cemiplimab + T-VEC	Metastatic	NA	PR	-	Skin	Responder	Primary
D pre	Male	N/A	Cutaneous	-	Pembrolizumab	Adjuvant	NA	NED	-	Lung	Non-response	Metastasis
E pre	Male	68	Cutaneous	-	Pembrolizumab	Adjuvant	NA	PD	-	Skin	Non-Responder	Primary
F pre	Male	50	Cutaneous	-	Pembrolizumab	Adjuvant	NA	PD	-	small bowel	Non-Responder	Metastasis
G pre	Male	85	Cutaneous	-	Nivolumab	Adjuvant	NA	PD	-	Superclavicular mass	Non-Responder	Metastasis
H pre	Male	59	Cutaneous	-	Nivolumab	Adjuvant	NA	NED	-	Skin	Responder	Metastasis
I pre	Male	53	Cutaneous	-	Pembrolizumab	Metastatic	NA	PD	-	Lung	Non-Responder	Metastasis
J pre	Male	N/A	Cutaneous-->Mucosal	-	Pembrolizumab	Metastatic	NA	PD	-	Sinonasal (ethmoid)	Non-Responder	Primary
K pre	Male	60	Cutaneous	-	Ipilimumab + Nivolumab	Metastatic	NA	PR	-	Skin	Responder	Primary
8 pre	Male	77	Mucosal	-	Pembrolizumab	Adjuvant	NA	PD	-	Perianal lesion	Non-Responder	Metastasis
9 pre	Female	56	Cutaneous	-	Nivolumab	Adjuvant	NA	PD	-	Brain, right frontal	Non-Responder	Metastasis
10 pre	Female	65	Mucosal	-	Nivolumab	Adjuvant	NA	PD	-	Left nasal polyp	Non-Responder	Primary
11 pre	Female	68	Cutaneous	-	Pembrolizumab	Metastatic	NA	CR	-	Lung Wedge Resection	Responder	Metastasis
12 pre	Male	72	Cutaneous	-	Pembrolizumab (2m) then T-VEC (2 months)	Adjuvant	NA	CR	-	Skin	Responder	Metastasis
13 pre	Female	33	Cutaneous	-	Ipilimumab + Nivolumab + Bevacizumab	Metastatic	NA	SD	-	Brain left frontal	Responder	Metastasis

Table S1. **Clinical annotations of the patient samples used in this study, related to Figure 7.** Clinical annotation of patients' biopsies taken after treatment (designated "post" in patient number) used in figure 7A,B and of patients' biopsies taken before treatment (designated "post" in patient number), used in Figure S7G,7B. All mIF stained samples. "Time to biopsy" refer to the time elapsed from the first day of treatment to biopsy collection. "Time to clinical outcome" refers to the time elapsed from the first day of treatment to clinical outcome determination. In blue writing, samples used in Figure 7B. Abbreviation: PD=Progressive disease. SD= stable disease. CR= complete response. NED=No evidence of disease. TVEC= Talimogene laherparepvec. ICB=immune checkpoint blockade.
